# The highly metastatic 4T1 breast carcinoma model possesses features of a hybrid epithelial/mesenchymal phenotype

**DOI:** 10.1242/dmm.050771

**Published:** 2024-09-04

**Authors:** Mary E. Herndon, Mitchell Ayers, Katherine N. Gibson-Corley, Michael K. Wendt, Lori L. Wallrath, Michael D. Henry, Christopher S. Stipp

**Affiliations:** ^1^ Department of Biology, University of Iowa, Iowa City, IA 52245, USA; ^2^ Department of Medicinal Chemistry and Molecular Pharmacology, Purdue University, West Lafayette, IN 47907, USA; ^3^ Department of Pathology, Microbiology and Immunology, Vanderbilt University Medical Center, Nashville, TN 37232, USA; ^4^ Holden Comprehensive Cancer Center, University of Iowa, Iowa City, IA 52242, USA; ^5^ Department of Internal Medicine, University of Iowa, Iowa City, IA 52242, USA; ^6^ Department of Biochemistry and Molecular Biology, University of Iowa, Iowa City, IA 52242, USA; ^7^ Department of Molecular Physiology and Biophysics, University of Iowa, Iowa City, IA 52242, USA

**Keywords:** E-cadherin, Breast cancer, Collective migration, Epithelial-mesenchymal transition, Metastasis

## Abstract

Epithelial-mesenchymal transitions (EMTs) are thought to promote metastasis via downregulation of E-cadherin (also known as Cdh1) and upregulation of mesenchymal markers such as N-cadherin (Cdh2) and vimentin (Vim). Contrary to this, E-cadherin is retained in many invasive carcinomas and promotes collective cell invasion. To investigate how E-cadherin regulates metastasis, we examined the highly metastatic, E-cadherin-positive murine 4T1 breast cancer model, together with the less metastatic, 4T1-related cell lines 4T07, 168FARN and 67NR. We found that 4T1 cells display a hybrid epithelial/mesenchymal phenotype with co-expression of epithelial and mesenchymal markers, whereas 4T07, 168FARN, and 67NR cells display progressively more mesenchymal phenotypes *in vitro* that relate inversely to their metastatic capacity *in vivo*. Using RNA interference and constitutive expression, we demonstrate that the expression level of E-cadherin does not determine 4T1 or 4T07 cell metastatic capacity in mice. Mechanistically, 4T1 cells possess highly dynamic, unstable cell-cell junctions and can undergo collective invasion without E-cadherin downregulation. However, 4T1 orthotopic tumors *in vivo* also contain subregions of EMT-like loss of E-cadherin. Thus, 4T1 cells function as a model for carcinomas with a hybrid epithelial/mesenchymal phenotype that promotes invasion and metastasis.

## Teaser

**Summary:** An intrinsically hybrid epithelial/mesenchymal phenotype confers dynamic remodeling of cell-cell junctions and an E-cadherin-indifferent metastatic capacity to the 4T1 model of stage IV breast cancer.

## INTRODUCTION

Epithelial-mesenchymal transitions (EMTs) have become a framework for explaining the metastatic capacity of carcinomas (tumors that arise in epithelial cell layers) (Brabletz et al., 2018). As extensively documented by foundational studies, EMT programs, which also play critical roles in development, wound healing and fibrosis, are governed by EMT transcription factors, including Zeb1, Zeb2, Twist (also known as Twist1) and Snail (or Snai1) (Thiery and Sleeman, 2006; Thiery et al., 2009; Hugo et al., 2007; Kalluri and Weinberg, 2009). Features of mesenchymal cancer cells produced by aberrant EMT activation in tumor progression include: (1) loss of the epithelial cell-cell adhesion protein, E-cadherin (Ecad or Cdh1), (2) upregulation of mesenchymal cadherins, such as N-cadherin (N-cad or Cdh2), (3) loss of epithelial cytokeratin intermediate filaments, (4) upregulation of the mesenchymal intermediate filament, vimentin (Vim), (5) disrupted cell-cell cohesion and loss of apical-basal polarity, and (6) increased cell migration and invasion into connective tissue, resulting in tumor cell dissociation from the primary tumor and dissemination to distant locations (Thiery and Sleeman, 2006; Thiery et al., 2009; Hugo et al., 2007; Kalluri and Weinberg, 2009). Subsequently, intriguing connections between EMT events and the acquisition of stem cell-like phenotypes were identified, with important implications for chemoresistance, recurrence and the capacity of disseminated tumor cells to generate macroscopic metastatic tumors (Brabletz et al., 2005; Mani et al., 2008; Blick et al., 2010; Lambert and Weinberg, 2021; Lytle et al., 2018). In addition, EMT-like states may also contribute to intermediate steps in the metastatic process, such as trans-endothelial migration of circulating tumor cells (Drake et al., 2009).

Early debates on the role of EMTs in metastasis often centered around the apparent lack of cancer cells with a definitive mesenchymal phenotype in human clinical carcinoma specimens (Tarin et al., 2005). A hypothesis for this paucity of mesenchymal cancer cells at both primary and metastatic tumor sites is that EMTs occur in a small subset of primary tumor cells and that a reverse, mesenchymal-epithelial transition (MET) occurs during outgrowth of macroscopic metastatic tumors (Thompson et al., 2005). This hypothesis has garnered considerable experimental support (Celià-Terrassa et al., 2012; Ocaña et al., 2012; Chaffer et al., 2006; Korpal et al., 2011; Tsai et al., 2012; Tran et al., 2014; Beerling et al., 2016; del Pozo et al., 2015). With the advent of the ability to capture circulating tumor cells and to profile gene expression via single-cell RNA sequencing, another hypothesis for the elusiveness of mesenchymal carcinoma cells is that a hybrid epithelial/mesenchymal (E/M) phenotype, also called quasi-mesenchymal phenotype, is linked to the acquisition of metastatic capacity that is more than that of a purely mesenchymal phenotype (Lambert and Weinberg, 2021; Jolly et al., 2022).

Uncertainties about the functional role of E-cadherin in regulating metastasis have mirrored the controversies about the roles of EMT and MET. On the one hand, E-cadherin has been described as the ‘keystone of the epithelial state’ (Scheel and Weinberg, 2012), such that loss of E-cadherin on its own is sufficient to trigger an EMT phenotype (Onder et al., 2008). Likewise, enforced E-cadherin expression can be sufficient to prevent metastatic outgrowth (Wendt et al., 2011; Mbalaviele et al., 1996). On the other hand, E-cadherin can play a key role in collective cell migration and invasion (Friedl and Mayor, 2017), and might function in some settings to promote the metastatic outgrowth of tumor cells *in vivo* (Padmanaban et al., 2019).

The immune-competent, murine 4T1 cell model of stage IV breast cancer has embodied both the progress and the lively controversy in the EMT field. For example, in one early study, highly metastatic 4T1 cells were used to reveal an important role of the EMT master regulator, Twist, in driving metastasis (Yang et al., 2004). However, subsequent studies have noted E-cadherin expression by 4T1 cells, despite their highly metastatic phenotype, and suggested possible pro-metastatic roles for E-cadherin in the 4T1 model system (Chu et al., 2013; Elisha et al., 2018).

In this study, we specifically sought to clarify the role of E-cadherin in the metastatic capacity of the widely used 4T1 model. Surprisingly, we found that downregulation of E-cadherin, sufficient to disrupt cell-cell cohesion, had minimal impact (positive or negative) on metastatic capacity. Furthermore, we found that despite the presence of E-cadherin, 4T1 epithelial cell-cell junctions are dynamic and unstable, leading to highly migratory phenotypes in two-dimensional (2D) and three-dimensional (3D) models of tumor cell migration and invasion. In an orthotopic *in vivo* mouse model, we found evidence of overt EMT-like events of 4T1 cell metastasis. Taken together, these findings are consistent with the highly aggressive phenotype in this widely used model of breast cancer progression. Moreover, our findings help to explain the mechanisms by which 4T1 cells have the capacity to manifest an aggressive hybrid E/M phenotype associated with metastasis.

## RESULTS

### E-cadherin expression levels do not determine metastatic capacity in the 4T1 breast carcinoma model, which displays hybrid E/M features

**Fig. 1.**
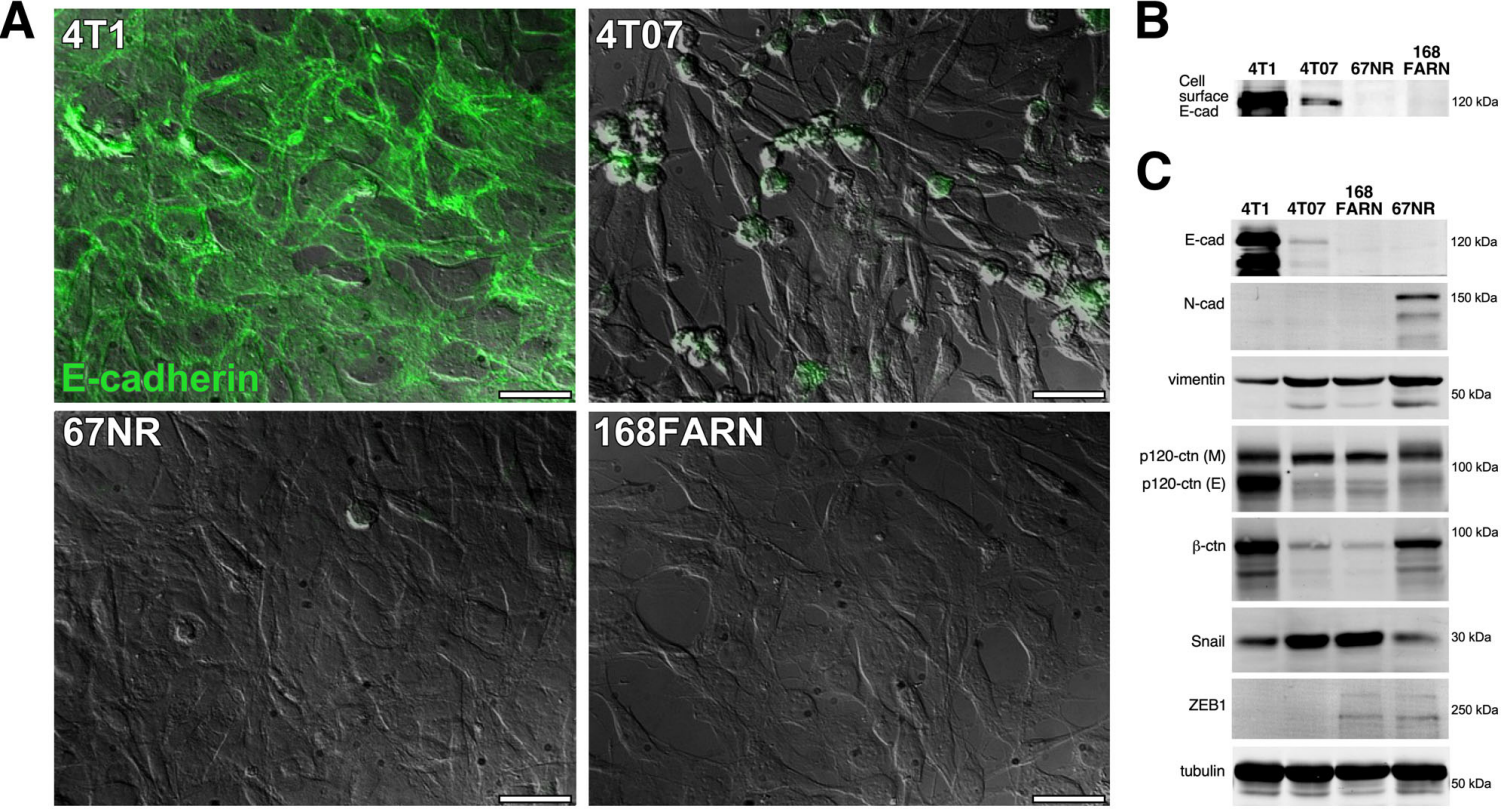
**E-cadherin expression correlates with increasing metastatic potential in 4T1 mouse mammary carcinoma cells and three co-isolated sublines.** (A) E-cadherin expression was examined by immunostaining in 4T1, 4T07, 67NR and 168FARN cells. Total E-cadherin staining (green) of permeabilized cells is shown superimposed over differential interference contrast (DIC) images. Images are representative of two independent experiments. Scale bars: 30 μm. (B) Cell surface-biotinylated cells were lysed with Brij 96 V and E-cadherin was immunoprecipitated from lysates of equivalent numbers of each cell type, followed by western blotting and detection of cell surface-labeled E-cadherin with NeutrAvidin using a LI-COR near-infrared gel imager. (C) Cell lysates were immunoblotted for E-cadherin (E-cad), N-cadherin (N-cad), vimentin, p120 catenin (p120-ctn), β-catenin (β-ctn), Snail, Zeb1, and tubulin. For p120 catenin, both mesenchymal p120-ctn (M) and epithelial p120-ctn (E) isoforms were detected in 4T1 cells. Blots in B,C are representative of three independent experiments.

Multiple studies have reported the seemingly paradoxical expression of E-cadherin in aggressive or metastatic 4T1 breast carcinoma cells (Korpal et al., 2011; Wendt et al., 2011; Chu et al., 2013; Elisha et al., 2018; Lou et al., 2008; Dykxhoorn et al., 2009; Wendt et al., 2014). Here, we directly evaluated the role of E-cadherin in either promoting or restraining metastasis in this model. We compared the relative levels of E-cadherin in 4T1 cells to E-cadherin levels in the less metastatic sublines (4T07, 168FARN and 67NR). These less metastatic cell lines were co-isolated with 4T1 from a spontaneous tumor in a Balb/C mouse (Aslakson and Miller, 1992). The 4T1 line featured prominent E-cadherin expression, whereas E-cadherin expression was reduced in the next most metastatic cell line, 4T07 (Fig. 1A). The two least metastatic cell lines, 168FARN and 67NR, had no detectable E-cadherin expression (Fig. 1A). Cell surface biotinylation followed by E-cadherin immunoprecipitation and detection using NeutrAvidin confirmed the immunostaining results (Fig. 1B).

To better characterize the epithelial versus mesenchymal phenotypes within this set of cells, we analyzed the expression of several additional EMT markers via western blotting (Fig. 1C). As anticipated, the 4T1 cells exhibited the most E-cadherin expression. The 4T07 cells showed a trace of E-cadherin expression, and no E-cadherin was detected in 168FARN or 67NR cells. The mesenchymal marker N-cadherin was only detected in 67NR cells, the least metastatic subline in the set. All four cell lines expressed vimentin, although vimentin levels were lower in 4T1 cells than in the other cell lines. In addition, all four cell lines expressed Snail and the higher-molecular-mass, mesenchymal isoform of p120 catenin (p120-ctn M; encoded by *Ctnnd1*) (Ohkubo and Ozawa, 2004). Interestingly, 4T1 cells, but not the other three cell lines, also expressed a substantial amount of the epithelial isoform of p120 catenin (p120-ctn E). 4T1 and 67NR cells expressed higher levels of the cadherin cytoplasmic partner, β-catenin (Ctnnb1), whereas β-catenin levels were reduced in 4T07 and 168FARN cells, in which E-cadherin and N-cadherin were reduced or absent. Lastly, the mesenchymal master regulator, Zeb1, was only detected in 168FARN and 67NR cells. Thus, with prominent expression of both epithelial and mesenchymal markers, 4T1 cells had the most overtly hybrid E/M phenotype, followed by the 4T07 cells, whereas the 168FARN and 67NR manifested a more mesenchymal phenotype.

It is possible that E-cadherin has the potential to suppress metastasis of primary tumor cells and/or promote collective invasion and the outgrowth of disseminated cells. To directly test the role of E-cadherin in regulating metastasis, we created 4T1 cells with either stable short hairpin RNA (shRNA)-induced knockdown of E-cadherin (Ecad-KD cells) or constitutive expression of E-cadherin possessing a C-terminal myc epitope tag (Ecad-myc cells). Importantly, prior studies have shown that the addition of a myc tag to the C-terminus of E-cadherin does not interfere with either E-cadherin localization or interactions with cytoplasmic protein partners such as β-catenin (Chitaev and Troyanovsky, 1998). By generating cells with reduced and enforced expression of E-cadherin, we could uncouple the expression levels of E-cadherin from transcriptional regulation by mesenchymal transcription factors.

**Fig. 2.**
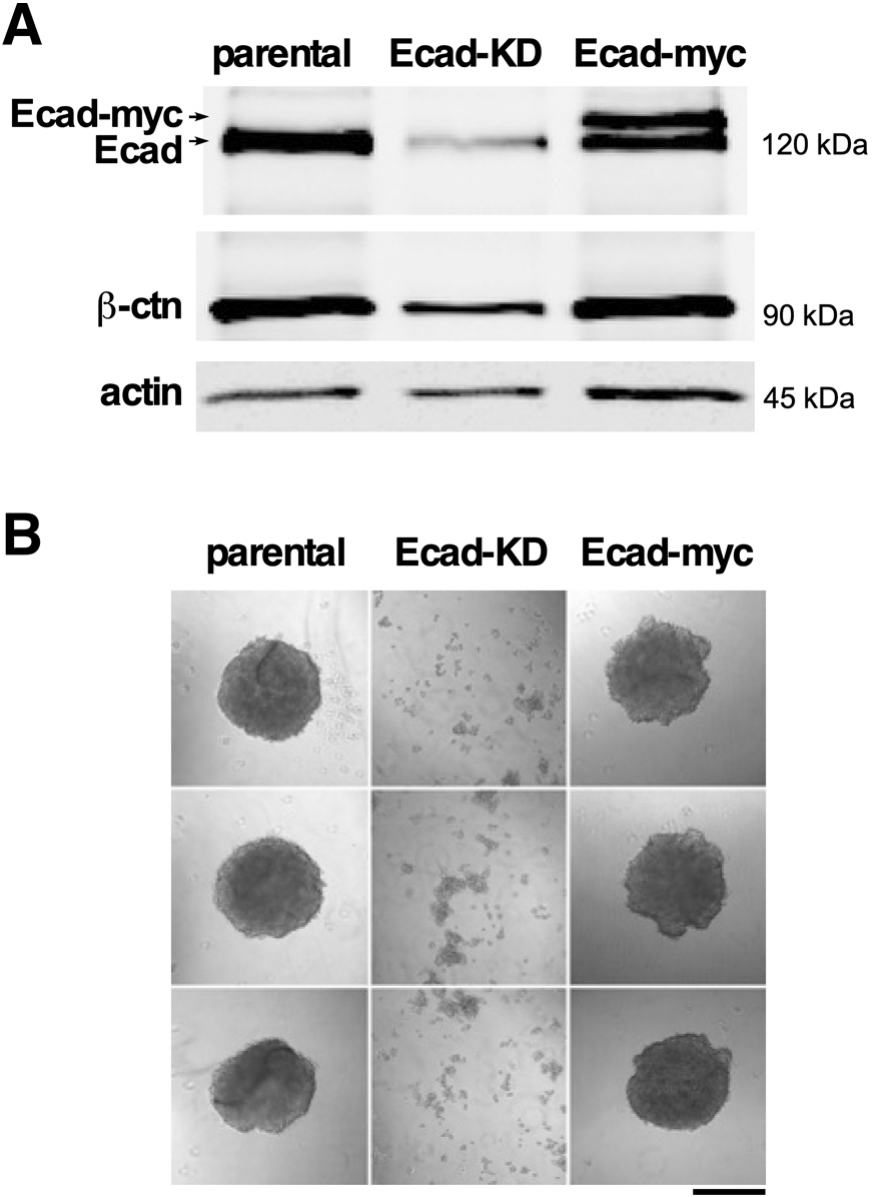
**Depletion of E-cadherin in 4T1 cells leads to a reduction in β-catenin and loss of E-cadherin function.** (A) Equivalent amounts of NP40 lysates of 4T1 cells (parental), Ecad-KD and Ecad-myc cells were loaded onto a SDS page gel. The proteins were size separated, transferred to membranes and incubated with antibodies to the proteins indicated. The primary antibodies were recognized by fluorescently labeled secondary antibodies and the membrane was imaged using a LI-COR blot system. The upper band in lane 3 corresponds to the anticipated size of myc-tagged E-cadherin. Blots are representative of two independent experiments. (B) Parental, Ecad-KD and Ecad-myc cell spheroids were recovered by pipetting (see Materials and Methods) and plated in 35 mm dishes for immediate photography. Knockdown of E-cadherin severely compromised the spheroid stability, indicating strong downregulation of cell-cell adhesion. Images are representative of two independent experiments. Scale bar: 500 μm.

Ecad-KD cells showed an ∼80% reduction in the level of E-cadherin compared to that in controls (Fig. 2A, lane 2). Interestingly, the level of β-catenin was also reduced in Ecad-KD cells compared to that in parental or Ecad-myc cells (Fig. 2A, lane 2). In some circumstances, the loss of E-cadherin might free the pool of E-cadherin-associated β-catenin to translocate to the nucleus and function as an EMT-promoting transcriptional coactivator (Tam and Weinberg, 2013; Lamouille et al., 2014), although this remains a topic of investigation (van der Wal and van Amerongen, 2020). However, we did not observe an obvious accumulation of nuclear β-catenin in Ecad-KD cells (Fig. S1). Collectively, these results suggest that, in the 4T1 model, the β-catenin protein liberated by the loss of E-cadherin is mostly degraded, rather than being stabilized as a nuclear transcription factor under standard cell culture conditions.

In cells that constitutively express Ecad-myc, surprisingly, the total level of E-cadherin remained nearly the same as that in the parental cells. However, approximately half of the endogenous E-cadherin appeared to be replaced with myc-tagged E-cadherin expressed from the constitutive retroviral promoter (Fig. 2A, lane 3). A possible explanation for the inability of our constitutive expression strategy to increase the total level of E-cadherin expression in 4T1 cells is that exogenous and endogenous E-cadherin compete for a limited pool of β-catenin, p120 catenin and/or vinculin (Vcl), each of which is required for optimal E-cadherin cell surface localization (Chen et al., 1999; Davis et al., 2003; Peng et al., 2010). Thus, endogenous cytoplasmic partners of E-cadherin might limit the total amount of E-cadherin that can be expressed in 4T1 cells. Regardless, Ecad-myc 4T1 cells expressed a substantial proportion of E-cadherin under the control of a constitutive promoter that should no longer be susceptible to transcriptional downregulation via mesenchymal transcription factors such as Zeb1. By contrast, Ecad-KD 4T1 cells lost a substantial proportion of E-cadherin prior to any induced EMT-like event. Thus, these cell lines were suitable for a functional test of the role of E-cadherin in 4T1 cell metastatic progression.

As a preliminary assessment of E-cadherin function in our cell lines, we performed spheroid-forming assays by culturing cells in wells coated with a non-adherent polyhydroxyethylmethacrylate (poly-HEMA) substrate. Upon recovery by gentle pipetting, the parental and Ecad-myc-expressing cells both yielded shear-resistant spheroids, whereas Ecad-KD cells could only be recovered as a mixture of single cells and small clumps (Fig. 2B). The loss of spheroid-forming capacity revealed a major functional impact of reducing the level of E-cadherin levels in the Ecad-KD cells.

**Fig. 3.**
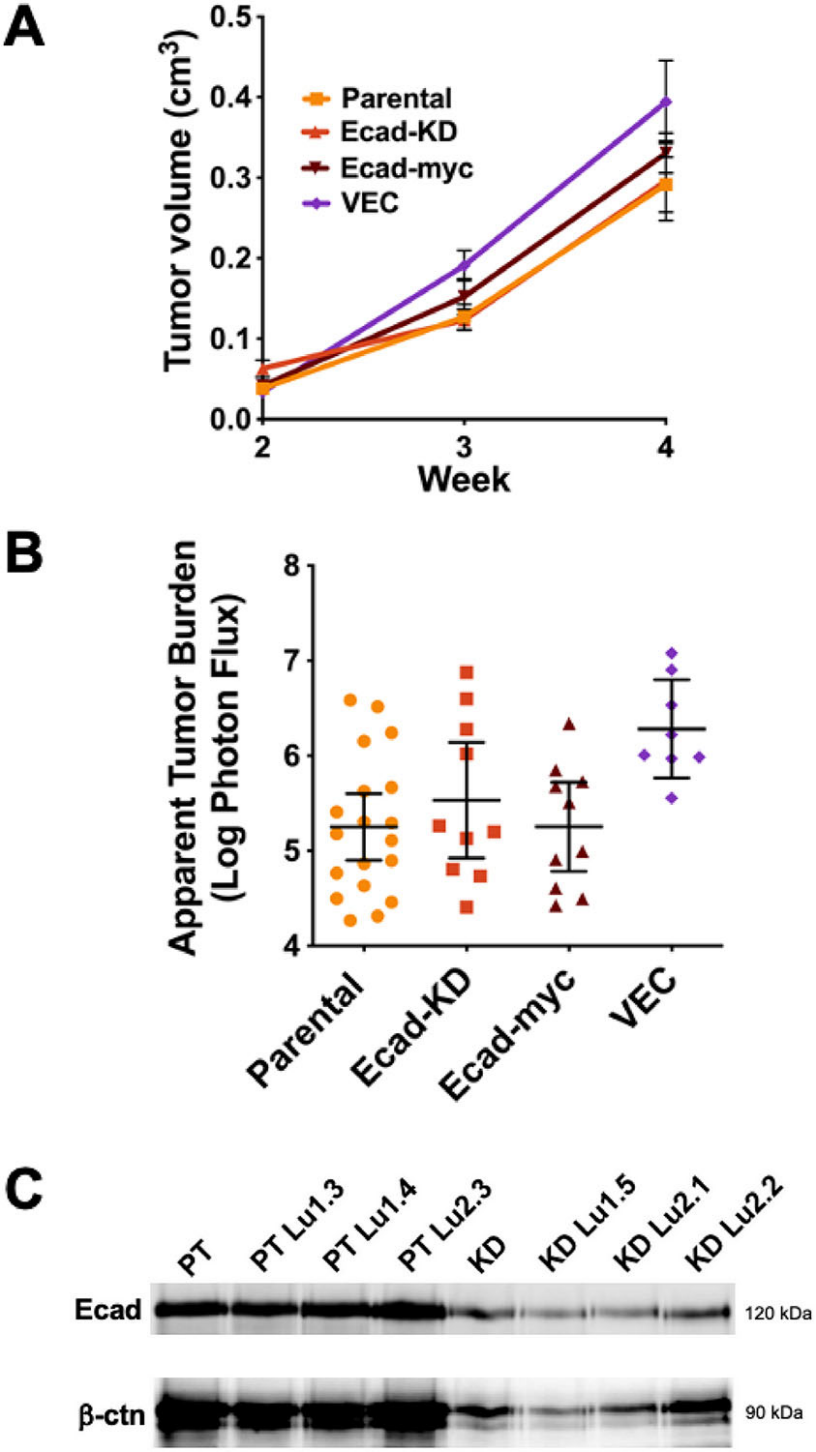
**The expression level of the E-cadherin–β-catenin complex does not determine the metastatic capacity in the 4T1 breast carcinoma model.** (A,B) The pooled results of two separate experiments, in which parental (*n*=10 mice) and Ecad-KD (*n*=10 mice) cells, or parental (*n*=10 mice), Ecad-myc (*n*=10 mice) and VEC (*n*=8 mice) cells were orthotopically implanted in Balb/C mice at 5×10^3^ cells/mouse. (A) Tumor growth at primary injection sites was monitored by caliper measurements over a 4-week period and the tumor volume was plotted. Bars show the mean±s.e.m. (B) The average apparent lung tumor burden for each group was measured at the assay endpoint, as described in the Materials and Methods. Bars show the mean±95% c.i. (C) Cells from lung metastatic tumors of parental (PT) and Ecad-KD (KD) cells implanted in mice were explanted as described in the Materials and Methods, and cell surface-labeled with biotin. E-cadherin levels were measured by immunoprecipitation from lysates of equivalent numbers of each cell type, followed by immunoblotting and detection with NeutrAvidin DyLight 800 (top panel). Co-precipitating β-catenin was detected with mouse anti-β-catenin, recognized by goat anti-mouse Alexa Fluor 680 (bottom panel). Imaging was performed using a LI-COR near-infrared gel imager. Three lung tumor explanted cell lines from both cell types are shown.

To directly test the extent to which E-cadherin expression influences metastasis in the 4T1 model, we orthotopically implanted parental, Ecad-KD, Ecad-myc and vector control cells in Balb/C mice and monitored tumor growth by caliper measurements. The four cell lines displayed similar growth rates at the primary site (Fig. 3A), and *ex vivo* bioluminescence imaging of lungs at the assay endpoint revealed no obvious changes in metastatic capacity upon either reduction or constitutive expression of E-cadherin (Fig. 3B). We considered the possibility that the similar lung metastatic outgrowth of Ecad-KD cells might reflect re-expression of E-cadherin by a population of cells escaping E-cadherin knockdown. However, measurements of E-cadherin levels and its associated partner, β-catenin, in protein extracts from metastatic tumor cells explanted from the lungs confirmed that knockdown of E-cadherin and the concomitant loss of associated β-catenin were maintained after *in vivo* passaging and recovery of Ecad-KD cells (Fig. 3C). Thus, the expression of the E-cadherin–β-catenin complex *in vitro* appears not to be a strong determinant of 4T1 cell metastatic capacity *in vivo*.

As an independent test of the role of E-cadherin in the metastatic process, we created 4T07 cells with E-cadherin overexpression (4T07-Ecad cells) (Fig. S2A). Compared to 4T07 cells lacking E-cadherin overexpression, 4T07-Ecad cells displayed virtually identical metastatic lung colonization upon tail vein injection in an experimental metastasis model (Fig. S2B). These results provide further evidence that E-cadherin expression on its own is not sufficient to determine the metastatic capacity in the 4T1 and 4T07 models.

### E-cadherin-based cell-cell junctions are perturbed in 4T1 cells

**Fig. 4.**
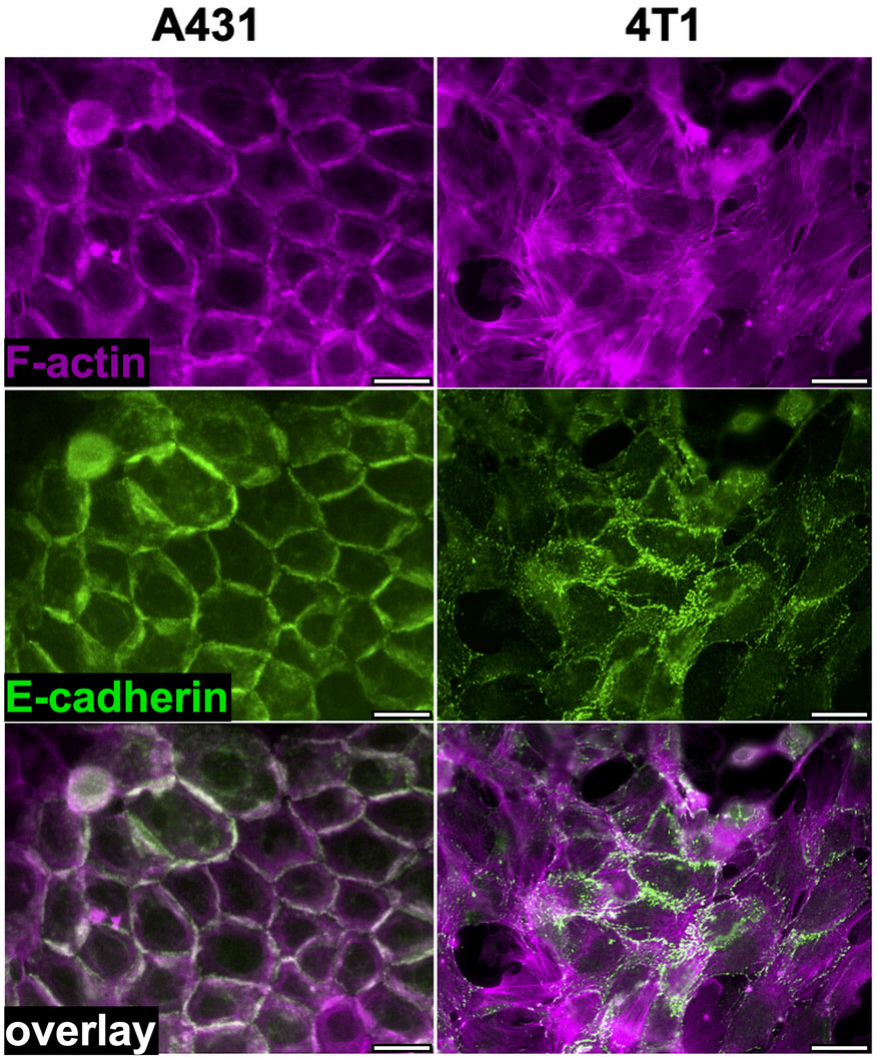
**E-cadherin-based cell-cell junctions are disorganized in 4T1 cells compared to those of the poorly metastatic A431 cell line.** A431 and 4T1 cells were stained for actin (Alexa Fluor 594-conjugated phalloidin; magenta) and E-cadherin (green fluorescence). Images are representative of three independent experiments. Scale bars: 30 μm.

**Fig. 5.**
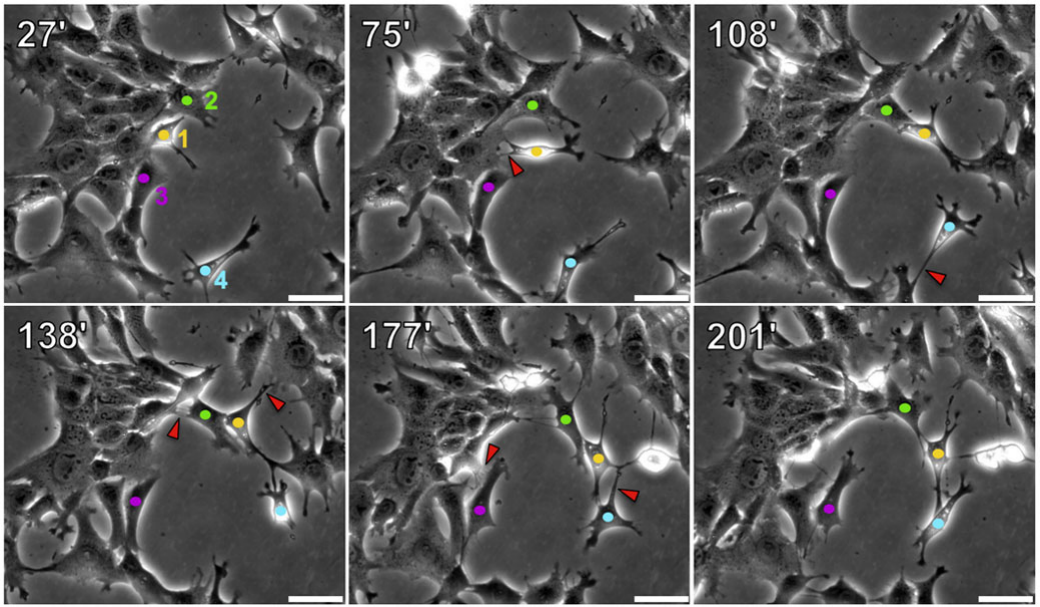
**4T1 cell-cell contacts are highly dynamic in 2D cultures.** 4T1 cells growing in 35 mm dishes were monitored by time-lapse microscopy for 6 h. The images show stills from Movie 1. Times of frames (in minutes) post start of the video are listed in the upper left corner of each panel. Four individual cells are marked in each frame with colored dots (cell 1 in yellow, cell 2 in green, cell 3 in magenta and cell 4 in teal). Red arrowheads mark cell-cell contacts that have separated in the subsequent time frame. Images are representative of two independent experiments. Scale bars: 50 μm.

The fact that *in vitro* E-cadherin expression levels do not influence 4T1 metastatic capacity *in vivo* suggests that E-cadherin function is altered in these cells. To examine this possibility, we compared the organization of E-cadherin-based cell-cell junctions in 4T1 cells to that of the poorly metastatic, E-cadherin-positive A431 carcinoma cells. In contrast to A431 cells, which displayed well-organized, circumferential cortical actin, much of the 4T1 actin cytoskeleton was in the form of actin cables, aligned perpendicular to cell-cell contacts (Fig. 4). Moreover, in 4T1 cells, E-cadherin-based junctions were jagged and irregular, compared to the orderly cell junctions in A431 cells (Fig. 4; see also Fig. S1). The organization of cell-cell junctions appeared similarly disturbed in both 4T1 parental and Ecad-myc cells, which both expressed similar levels of total E-cadherin (Fig. S3). In conjunction with their poor organization, 4T1 cell junctions also displayed highly dynamic remodeling. Time-lapse video microscopy revealed that 4T1 cells constantly broke and reformed cell-cell contacts, even over relatively short observation periods (Fig. 5, Movie 1), as also reported by Elisha et al. (2018). Thus, although 4T1 cells can form E-cadherin-dependent, shear-resistant spheroids (Fig. 2B), they are able to rapidly remodel their cell-cell contacts in 2D cultures.

**Fig. 6.**
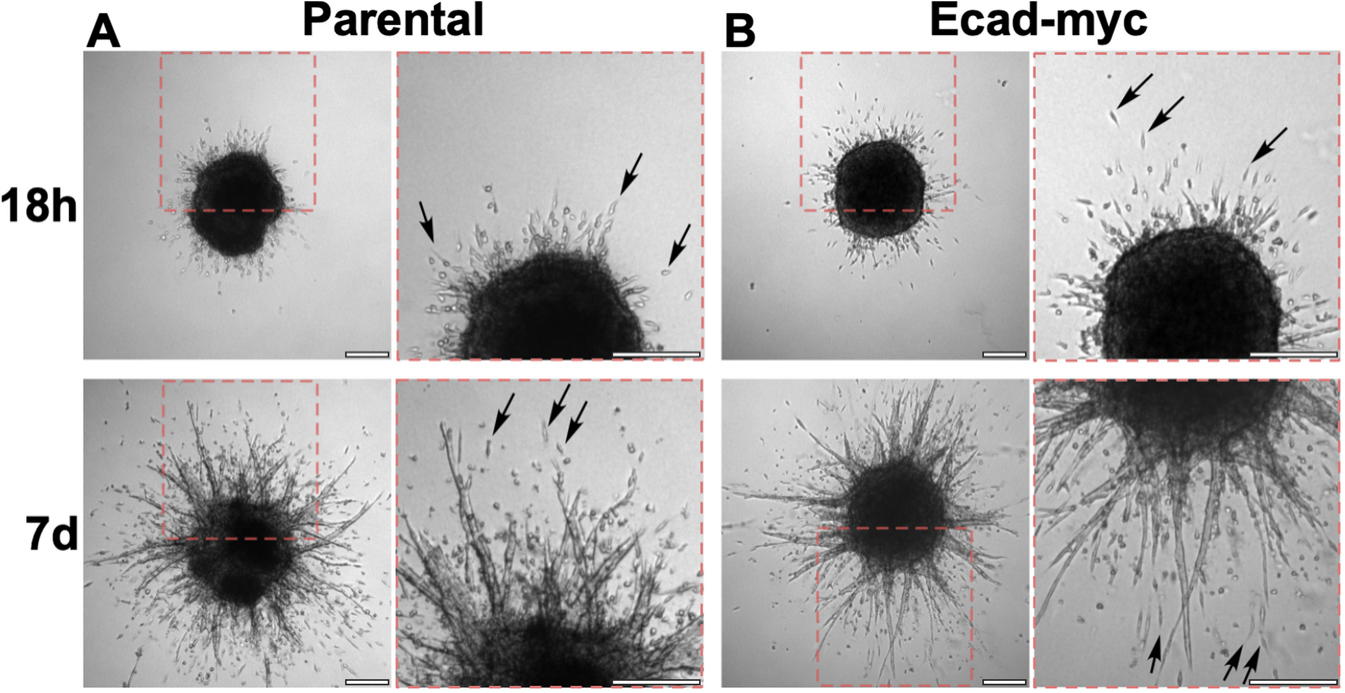
**Parental and Ecad-myc 4T1 cell spheroids display extensive invasion of single cells and multicellular structures in 3D collagen.** Spheroids of parental (A) and Ecad-myc (B) 4T1 cells were prepared as described in the Materials and Methods. After embedding in 3D collagen, micrographs of full spheroids were taken at 18 h and 7 days. Dashed red boxes in the left panels indicate the regions magnified in the adjacent panels. Arrows point to examples of single cells that have detached from the spheroids during invasion. Images are representative of two independent experiments. Scale bars: 250 μm.

To examine the stability of 4T1 cell-cell junctions in 3D cultures, we created spheroids as in Fig. 2 and embedded them in a 3D collagen matrix. After overnight culture, both parental and Ecad-myc cell spheroids were surrounded by a halo of single cells and small groups of cells that had invaded into the collagen matrix (Fig. 6A,B). By 1 week post embedding, both types of spheroids displayed extensive invasion, with single cells and multicellular structures emanating from the spheroid (Fig. 6A,B). Ecad-KD cells were not used in this assay as intact Ecad-KD cell spheroids could not be recovered (Fig. 2B).

**Fig. 7.**
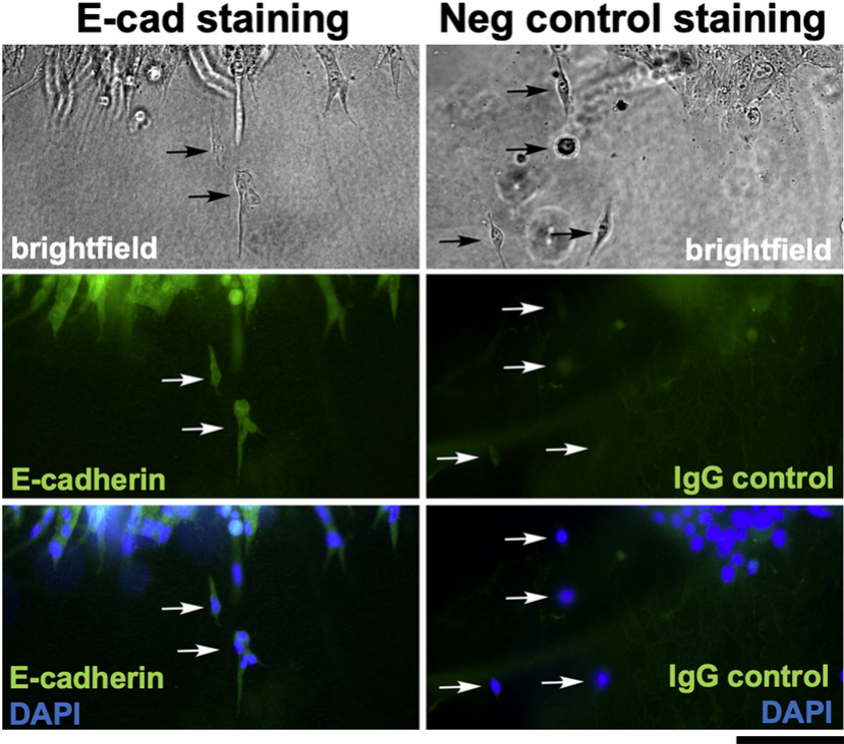
**E-cadherin is retained in single cells and clusters of cells emerging from 4T1 cell spheroids embedded in 3D collagen.** The spheroid depicted in Fig. S4 and Movies 2 and 3 was fixed after the 40-h time-lapse experiment and stained with an anti-E-cadherin antibody. Another spheroid from a parallel experiment was fixed and stained with a mouse IgG isotype negative control antibody. For both samples, cell nuclei were visualized with DAPI, and the edges of the spheroids were imaged by brightfield and fluorescence microscopy. Arrows point to single cells or small groups of cells that have emerged from the spheroids. These results demonstrate that cells and cell clusters invading into the 3D collagen matrix either retained E-cadherin expression throughout the invasion process or rapidly re-expressed E-cadherin upon dissociation from the spheroid. Images are from one experiment in which spheroid invasion was monitored by time-lapse imaging followed by immunostaining. Scale bar: 100 μm.

The ability of individual 4T1 cells to break free from a spheroid and invade a 3D collagen matrix within 18 h could be explained by an EMT-like event, in which the most invasive cells downregulated E-cadherin. Therefore, we investigated the timing of the onset of invasion of parental 4T1 cells by time-lapse video microscopy. As early as 2 h after embedding, invasive cells oriented towards the collagen matrix were evident at the spheroid boundary (Fig. S4). By as early as 5 h after embedding, the first singly invading cells had fully emerged from the spheroid (Fig. S4; see also Movie 2). The process by which single cells and small groups of cells emerged from the spheroid was monitored in detail through video recordings (Movie 3, showing a zoomed-in field of Movie 2). Invading cells appeared to overcome significant traction forces exerted by the spheroid, which often appeared to pull emerging cells back into the spheroid. Eventually, individual cells or small clusters of cells freed themselves from the spheroid, often emerging from the tips of strands of collectively invading cells (Movie 3), such as the invasive structures described by Cheung et al. (2013) in tumors and organoids from the mouse mammary tumor virus-polyoma middle tumor antigen (MMTV-PyMT) breast cancer model, as well as primary human breast tumor explants in a 3D collagen I matrix. To evaluate E-cadherin expression in the invading cells, we fixed the spheroid and immunostained for E-cadherin (Fig. 7, Movies 2 and 3). E-cadherin expression was evident in both single cells and small groups of cells invading in contact with each other (Fig. 7). Negative control (IgG) staining of another spheroid showed only a low background of non-specific signal (Fig. 7). Thus, a loss of E-cadherin was either extremely transient or unnecessary for cell escape and invasion in this model. Collectively, these observations suggest that the ability of E-cadherin to enforce key features of an epithelial phenotype is disrupted in 4T1 cells, facilitating their invasion without loss of E-cadherin.

### A hybrid E/M phenotype and EMT-like changes in subregions of a tumor may contribute to 4T1 cell metastasis *in vivo*

**Fig. 8.**
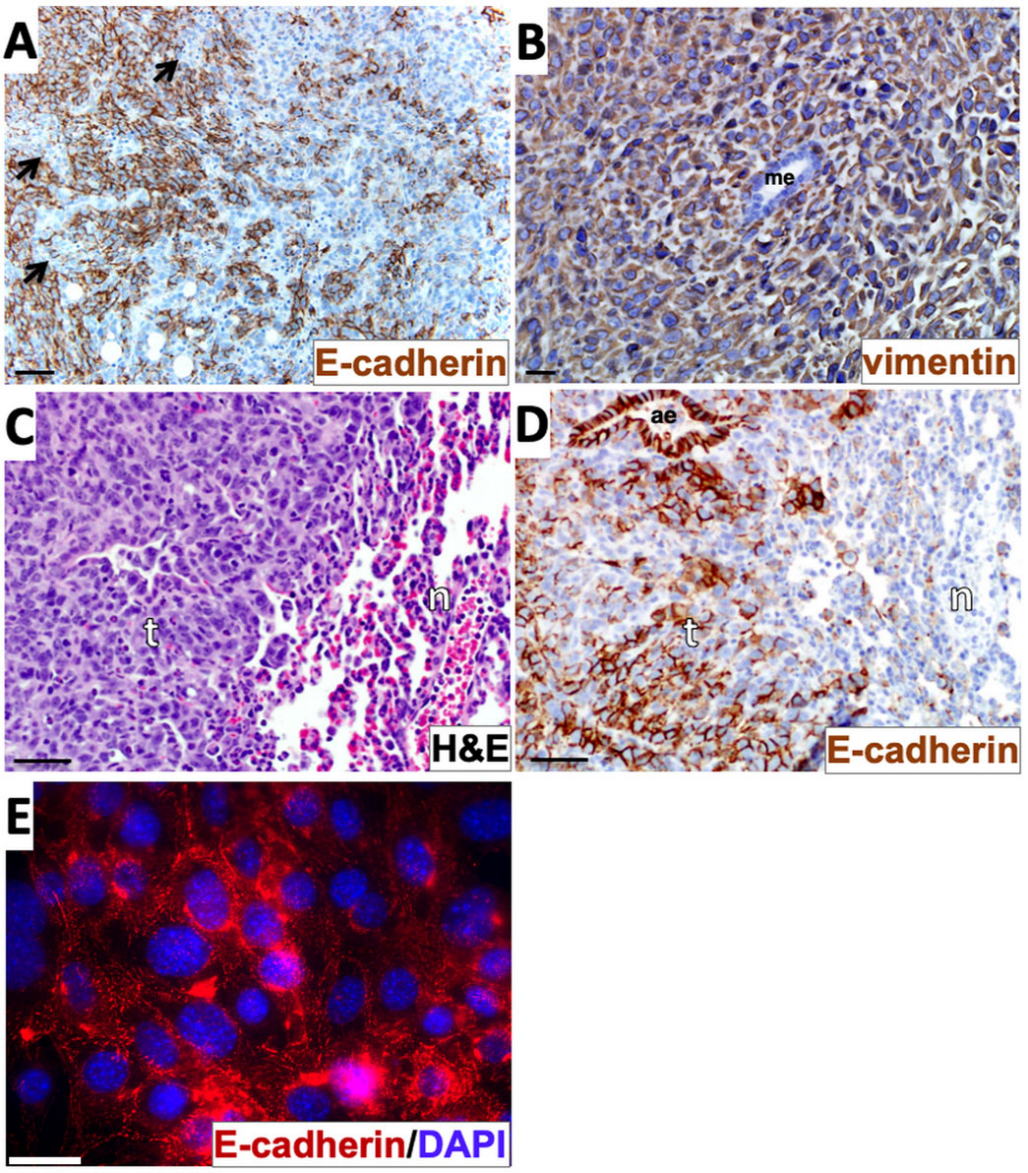
**Evidence of a hybrid epithelial/mesenchymal phenotype and epithelial-mesenchymal transition-like events in the 4T1 model.** (A) Primary tumor tissue was stained with E-cadherin immunohistochemistry (brown) and counterstained with Hematoxylin. Arrows indicate E-cadherin-negative zones within an overall E-cadherin-positive sector of the tumor. (B) Primary tumor tissue was stained with vimentin immunohistochemistry (brown) and counterstained with Hematoxylin. (C,D) Tissue from lung metastatic tumors were stained with Hematoxylin and Eosin (H&E) (C) or with E-cadherin immunohistochemistry and Hematoxylin counterstain (D). (E) Cells explanted from a 4T1 metastatic lung tumor as described in the Materials and Methods were immunostained with anti-E-cadherin (red) and labelled with DAPI (blue). Images in A-D are representative of three different tumor sections taken from one *in vivo* experiment. The image in E represents cells from one lung metastasis explanted *in vitro*. ae, airway epithelium; n, normal lung tissue; me, mammary epithelium; t, tumor tissue. Scale bars: 50 µm (A,C,D); 20 µm (B); 30 μm (E).

To further investigate the hybrid E/M phenotype of 4T1 cells *in vivo*, we immunostained primary tumors and lung metastatic for E-cadherin. We observed areas in which a subset of tumor cells appeared E-cadherin negative, even when juxtaposed to areas of E-cadherin-positive cells (Fig. 8A). We also analyzed 4T1 tumors by immunostaining for the mesenchymal marker vimentin. (Fig. 8B). Unlike E-cadherin, which was variably expressed, vimentin expression appeared uniform throughout 4T1 primary tumors, similar to a prior report (Liu et al., 2019). By contrast, vimentin staining was absent from an internal negative control of mammary epithelial cells captured in the same tissue slice (Fig. 8B). Thus, primary 4T1 cell tumors contain populations of cells that express both E-cadherin and vimentin, as well as populations that lack E-cadherin and express vimentin.

To determine whether this heterogeneity in E-cadherin expression also exists in metastases, lung metastatic tumor tissue (Fig. 8C) was immunostained with an antibody to E-cadherin. E-cadherin expression was variable within lung metastatic tumor tissue, but clearly present within the nearby airway epithelial cells serving as an internal positive control within the same tissue slice (Fig. 8D). Thus, as we observed in primary tumors, both E-cadherin-positive and -negative cells were found in metastatic tumors. By contrast, when lung metastatic cells were recovered and cultured *in vitro* (selecting for tumor cells based on G418 resistance, a feature of the luciferase expression vector), universal E-cadherin expression was observed by the time a bulk population of explanted tumor cells was recovered (Fig. 8E). This suggests that retention or re-expression of E-cadherin is a feature of cells that can grow out more efficiently in culture following explantation. Taken together, these data support a scenario in which E-cadherin is downregulated in a subset of cells in 4T1 primary tumors, which are already composed of cells with a hybrid E/M phenotype that could facilitate collective invasion and dissemination without E-cadherin downregulation. This combination of altered cell adhesion proteins and hybrid cellular identity is a feature consistent with the aggressive metastatic behavior of the 4T1 carcinoma model.

## DISCUSSION

E-cadherin has classically been viewed as a metastasis suppressor due to its ability to enforce a sessile, epithelial phenotype with strong, organized cell-cell junctions and attenuation of β-catenin signaling (Scheel and Weinberg, 2012; Onder et al., 2008; Wendt et al., 2011; Mbalaviele et al., 1996; Tam and Weinberg, 2013; Lamouille et al., 2014; van der Wal and van Amerongen, 2020). Triggering an EMT can result in downregulation of E-cadherin, promoting tumor cell invasion and metastasis by releasing the anti-migratory constraints of cell-cell contact. Conversely, at least in some settings, MET may be required at the metastatic site to facilitate macroscopic tumor outgrowth (Celià-Terrassa et al., 2012; Ocaña et al., 2012; Chaffer et al., 2006; Korpal et al., 2011; Tsai et al., 2012; Tran et al., 2014; Beerling et al., 2016; del Pozo et al., 2015). E-cadherin expression is often used as a marker to monitor EMT and MET events; however, the extent to which E-cadherin itself is required to restrain invasion at the primary tumor site or to promote growth upon MET at a secondary tumor site remains the subject of ongoing investigation. Here, we found that neither depletion nor constitutive expression of E-cadherin had an obvious impact on 4T1 tumor growth or metastasis, or 4T07 metastatic colonization, adding to the evidence that acquiring metastatic and non-metastatic properties are complex processes that rely on more than E-cadherin as a switch.

Our results contrast with those of Chu et al. (2013), who reported that RNAi silencing of E-cadherin in the SUM149 and Mary-X inflammatory breast cancer cell lines abrogated growth *in vivo*. Depleting E-cadherin by RNAi in 4T1 cells was also reported to block primary tumor growth in this study. One possible explanation for this discrepancy is that our 4T1 Ecad-KD cells were maintained as a polyclonal population, whereas Chu et al. (2013) isolated a subclone, which potentially might have been more strongly dependent on E-cadherin for growth than the bulk 4T1 cell population.

Our results also contrast with those of Elisha et al. (2018), who reported a modest reduction in 4T1 cell lung metastatic growth upon E-cadherin knockdown, when observed at early time points (∼1-2 weeks after tail vein or orthotopic injection). In our study, both E-cadherin-depleted and Ecad-myc-expressing cells displayed a similar growth rate compared to that of control cells at the primary site and no obvious difference in lung colonization at a later time point (after ∼4 weeks). Our observations agree with a preliminary report that complete deletion of E-cadherin via CRISPR gene editing had no obvious impact on 4T1 cell metastatic capacity (Spencer et al., 2015). In addition, our results agree with Elisha et al. (2018) with regards to the dynamic nature of 4T1 cell-cell junctions and the possibility that modes of collective cell migration involving E-cadherin contribute to 4T1 cell metastasis. On balance, the available data suggest that E-cadherin expression in the bulk 4T1 cell population has only a modest direct impact on the metastatic capacity of the cells, at least when measured at later time points, rendering the cells largely E-cadherin indifferent regarding the ultimate outcome of disease progression using this model. Our ability to deplete E-cadherin by ∼80% without blocking macroscopic outgrowth may reflect either that the ∼20% residual E-cadherin expression in the E-cadherin-depleted cells was sufficient to support outgrowth, or that expression of E-cadherin by itself is not the critical aspect of the epithelial phenotype that serves to promote macroscopic outgrowth.

Although 4T1 cells retain E-cadherin expression *in vitro* and form E-cadherin-dependent, shear-resistant spheroids, immunostaining and time-lapse video microscopy revealed that 4T1 cell-cell junctions are disorganized and highly dynamic (Figs 4 and 5, Movies 1-3). The ability of E-cadherin-positive 4T1 cells to dissociate from tumor spheroids is reminiscent of the mechanical disruption of cell-cell junctions upon treatment of Madin–Darby canine kidney epithelial (MDCK) cells with hepatocyte growth factor (HGF) (de Rooij et al., 2005). In this study, HGF triggered MDCK cell scattering without downregulation of E-cadherin. Instead, HGF activated a contractile actin cytoskeleton, with actin bundles terminating orthogonally at cell-cell boundaries, similar to what we observed in 4T1 cells. The increased actomyosin contractility in response to HGF caused MDCK cell-cell junctions to be pulled apart, with no detectable reduction in cell surface E-cadherin expression. We propose that 4T1 cell junctions are constitutively disorganized and dynamic, contributing to their invasive and metastatic phenotype, despite their retention of E-cadherin expression.

The 4T1 murine breast carcinoma is a widely utilized model of metastatic breast cancer because it is one of few cell lines that features rapid, robust spontaneous metastasis from an orthotopic site in an immunocompetent host. Prior studies have reported 4T1 EMT events *in vivo*, and the isolation of circulating and disseminated 4T1 cell sublines with both mesenchymal and hybrid E/M molecular phenotypes (Wendt et al., 2014; Liu et al., 2019). Our new results solidify that 4T1 cells display features of an intrinsic hybrid E/M phenotype, even under basal cell culture conditions *in vitro*. Retrospectively, the functional significance of 4T1 cells possessing a hybrid E/M phenotype is supported by several additional studies, which, at the time that they were performed, were not directly aimed at establishing an E/M phenotype in these cells (Table S2). A landmark study describing the expression of the EMT-inducing transcription factor Twist in 4T1 cells helped to advance the concept of EMT as a critical determinant of metastasis (Yang et al., 2004). In addition to Twist, the EMT-promoting transcription factor Zeppo1 (also known as Znf703) may also contribute to 4T1 cell metastasis (Slorach et al., 2011). However, we and others noted retention of E-cadherin and the epithelial-specific splicing factor Esrp1 in the 4T1 model (Korpal et al., 2011; Chu et al., 2013; Elisha et al., 2018; Spencer et al., 2015; Yae et al., 2012). Analyses of microRNA expression in the 4T1 cell system have also revealed co-expression of both pro-epithelial miRNAs (miR-200 family and miR-155) and pro-mesenchymal miRNAs (miR-9 and miR10b) (Korpal et al., 2011; Dykxhoorn et al., 2009; Ma et al., 2010a,b; Xiang et al., 2011). The co-expression of epithelial and mesenchymal master regulators may be critical for the aggressive phenotype of 4T1 cells as depleting Esrp1, Twist, Zeppo1, miR-9 or miR-10b all suppress 4T1 metastasis (Yang et al., 2004; Slorach et al., 2011; Yae et al., 2012; Ma et al., 2010a,b), whereas overexpressing miR-200 family members promotes metastasis of 4T07 breast carcinoma cells, which normally fail to form macroscopic colonies in the lung (Korpal et al., 2011; Dykxhoorn et al., 2009). Moreover, altering the expression of the ‘phenotypic stability factors’ Ovol2 or Grhl2, both of which may help to enforce a stable E/M phenotype (Jia et al., 2019), also reduced metastatic capacity in the 4T1 model (Wu et al., 2017; Wang et al., 2023). Lastly, Snail has been proposed as an EMT transcription factor that promotes a hybrid E/M state and increased metastatic capacity (Kröger et al., 2019), and manipulating Snail protein expression by forced expression or RNAi-mediated knockdown of the ubiquitin E3 ligase Fbxo11, which targets Snail, provided evidence that Snail expression promotes 4T1 cell metastasis (Zheng et al., 2014). Collectively, these multiple, independent studies strongly support the view that the 4T1 model can manifest a hybrid E/M state of metastatic breast cancer.

An interesting aspect of the hybrid phenotype of 4T1 cells is their co-expression of epithelial and mesenchymal p120 catenin isoforms. Intriguingly, Zeppo1, which promotes 4T1 cell metastasis, also promoted a shift in p120 catenin expression from the epithelial isoform to the mesenchymal isoform in non-tumorigenic mammary epithelial cells cultured in 3D (Slorach et al., 2011). Inducers of a mesenchymal phenotype can promote dramatic morphological changes even in the ongoing presence of E-cadherin. For example, TGF-β-treated NMuMG mammary epithelial cells undergo a profound morphological transition with a loss of organized cell junctions that precedes the loss of E-cadherin cell surface expression by at least 2 days (Maeda et al., 2005). In this study from Wheelock and colleagues, molecular changes in response to TGF-β treatment included disruption of the normal cortical actin cytoskeleton and formation of trans-cellular stress fibers, similar to the arrangement of the actin cytoskeleton in 4T1 cells. These changes occurred early, despite the initial maintenance of E-cadherin and the continued association of E-cadherin with its partners, α- and β-catenin (Maeda et al., 2005). We suggest that, in a similar manner, a constitutive, partially mesenchymal phenotype overrides the ability of E-cadherin to maintain organized adherens junctions in 4T1 cells.

If 4T1 cells possess a constitutive, partially mesenchymal phenotype that can override the ability of E-cadherin to restrain detachment and invasion, one might posit that no further EMT-like events would be required to facilitate their metastasis. However, analysis of cells *in vivo* suggests additional layers of complexity. We detected the maintenance of E-cadherin-positive 4T1 cells *in vivo*, as previously reported (Lou et al., 2008); however, close inspection revealed regions of tumor cells with reduced levels of E-cadherin, which has also been reported in the 4T1 model (Wendt et al., 2014). Staining of E-cadherin in metastatic colonies revealed a similar picture, with mixed E-cadherin-positive and -negative cells (Fig. 8). Overall, our data suggest that localized E-cadherin downregulation, overlaid onto a phenotype that is already partially mesenchymal, contributes to the aggressive nature of the 4T1 breast carcinoma *in vivo*. As such, the 4T1 model can serve as an experimental platform to explore potential treatment strategies for invasive carcinomas that retain E-cadherin expression. Such strategies may include restoring the ability of E-cadherin to suppress invasion, as suggested by a study using an E-cadherin-activating antibody in the 4T1 model (Na et al., 2020). Conversely, a better understanding of how epithelial master regulators such as miR-200 and miR-155 can sometimes function to promote metastatic outgrowth may lead to novel strategies for selectively targeting the pro-metastatic functions associated with an epithelial phenotype.

## MATERIALS AND METHODS

### Antibodies

Primary antibodies used in the study are listed in Table S1. Fluorescently labeled secondary antibodies for immunostaining were Alexa Fluor 488 and Alexa Fluor 594 goat anti-mouse IgGs (1:400, A-11029 and A-11032, Thermo Fisher Scientific). Fluorescently labeled secondary antibodies for western blot analyses were DyLight 680 goat anti-mouse and DyLight 800 goat anti-rabbit IgGs (1:10,000, 610-144-121 and 610-145-122, Rockland Immunochemicals, Pottstown, PA, USA).

### Cell culture, RNA interference and retroviral transduction

All cell culture reagents and antibiotics were from Thermo Fisher Scientific, except where otherwise specified. The 4T1 cell line was obtained from the American Type Culture Collection (ATCC; Manassas, VA, USA), and the 4T07, 67NR and 168FARN mouse mammary carcinoma lines were gifts from Fred Miller (Wayne State University, Detroit, MI, USA). These cell lines were tested to be free of mycoplasma prior to initiating the study. Collectively, these cells were cultured in RPMI medium supplemented with 1× non-essential amino acids. A431 epithelial carcinoma cells (ATCC) and GP2-293 cells (Takara Bio USA; San Jose, CA, USA) were cultured in high-glucose Dulbecco's modified Eagle medium. All cultures were supplemented with 2 mM L-glutamine, 100 U/ml penicillin, 100 µg/ml streptomycin and 10% fetal bovine serum (Valley Biomedical, Winchester, VA, USA). Serum-free medium for the mammary carcinoma lines was RPMI supplemented with 1× non-essential amino acids, 25 mM HEPES and 5 mg/ml cell culture-grade bovine serum albumin (A1470, Sigma-Aldrich, St. Louis, MO, USA). Antibiotics used for selection included G418, zeocin and puromycin (Gold Biotechnology, St. Louis, MO, USA).

To facilitate monitoring of tumor growth *in vivo*, 4T1 cells were transduced with a luciferase cDNA cloned into the pQCXIN retroviral expression vector (Takara Bio USA), selected with 0.5 mg/ml G418, and maintained in 0.1 mg/ml G418. This parental cell line (4T1-lucIN) was used to create E-cadherin knockdown cells (Ecad-KD), cells constitutively expressing myc-tagged E-cadherin (Ecad-myc) and empty vector control (VEC) cells.

For RNAi, double-stranded oligonucleotides encoding shRNAs targeting the mouse E-cadherin mRNA were annealed and then cloned into the pSIREN-RetroQ retroviral vector (BD Biosciences, Franklin Lakes, NJ, USA). The shRNA targeting sequence for E-cadherin was 5′-TACATCCTTCATGTGAGAGTG-3′. This construct was co-transfected with the pVSV-G retroviral coat protein expression vector (Takara Bio USA) into GP2-293 packaging cells (Takara Bio USA) using Effectene (Qiagen Sciences, Germantown, MD, USA). At 24 h and again at 48 h post transfection, virus-containing medium was collected, filtered using a 0.45 µm filter, supplemented with 4 µg/ml polybrene (Sigma-Aldrich), and then used to transduce 4T1-lucIN cells. Stable transductants (Ecad-KD cells) were selected with 2 µg/ml puromycin, maintained in 1 µg/ml puromycin and 0.1 mg/ml G418, and sorted by flow cytometry to obtain a polyclonal population with greatly reduced E-cadherin expression. To sort cells with low E-cadherin expression, cells were harvested with PBS containing 1 mM EDTA (PBS/EDTA) lacking trypsin, as E-cadherin epitopes are trypsin sensitive. Cells were rinsed and resuspended in sterile 10% goat serum in PBS before staining with E-cadherin primary antibody, followed by staining with fluorescent secondary antibodies.

To create Ecad-myc cells, mouse E-cadherin cDNA [image clone 3002385; Open Biosystems (acquired by Thermo Fisher Scientific)] was cloned into an LXIZ retroviral vector engineered to contain a high-affinity C-terminal myc tag (Hilpert et al., 2001). The same LXIZ vector lacking an insert was used to create empty vector control cells (VEC). These constructs were transfected into GP2-293 cells, then used to transduce 4T1-lucIN cells using the method above. Stable transductants were selected with 0.3 mg/ml zeocin, and then maintained in 0.1 mg/ml zeocin and 0.1 mg/ml G418. To create 4T07 E-cadherin-overexpressing cells, the NF-κB 5× promoter of the pNifty2-N-Fluc-Zeo vector (InVivoGen, San Diego, CA, USA) was replaced with the CMV promoter from pcDNA3.1 (Thermo Fisher Scientific). The resulting vector was transfected into 4T07 cells, and cells with stable expression were selected using zeocin. These cells were subsequently transduced with retroviral particles constructed using pWZL, encoding full length E-cadherin (Addgene, #18804). Following transduction, stable expression was selected for using blasticidin.

### Immunoprecipitation and western blotting

For immunoprecipitation of cell surface-labeled E-cadherin, cells were cell surface biotinylated with 0.1 mg/ml Sulfo-NHS-LC Biotin (Thermo Fisher Scientific) in HEPES-buffered saline with magnesium chloride (HBSM; 20 mM HEPES pH 7.2, 150 mM NaCl, 5 mM MgCl_2_) for 1 h at room temperature and then rinsed three times with HBSM prior to lysis. Cells were lysed in PBS with 1% detergent, protease inhibitors (2 mM PMSF, 10 µg/ml aprotinin, 5 µg/ml leupeptin and 5 µg/ml E-64) and HALT phosphatase inhibitor (Thermo Fisher Scientific). Detergents were NP-40 or Brij 96 V (both from Sigma-Aldrich). Lysates were clarified, protein concentrations measured using the Red 660 Protein Assay (G-Biosciences, St. Louis, MO, USA), then lysate concentrations were normalized. Immune complexes, composed of anti-E-cadherin antibody combined with Protein G Agarose (Thermo Fisher Scientific), were collected, separated by SDS-PAGE and transferred to nitrocellulose membranes. The resulting membrane was blocked with Intercept TBS (LI-COR Biosciences, Lincoln, NE, USA), incubated with NeutrAvidin DyLight 800 (Thermo Fisher Scientific) and analyzed with a LI-COR near-infrared fluorescence imager. In some experiments, co-precipitating β-catenin was detected by immunoblotting.

For analysis of epithelial and mesenchymal markers, cells were lysed in SDS-PAGE sample buffer and protein concentrations were measured via the Red 660 assay. A total of 30 µg of protein/lane was loaded, separated by SDS-PAGE and transferred to Immobilon-FL membranes (Millipore Sigma, Burlington, MA, USA). After incubation with primary antibodies (see Table S1), followed by incubation with fluorescent secondary antibodies, the membranes were analyzed with an Odyssey imager (LI-COR).

### Immunostaining cultured cells and spheroids

4T1 and related cell lines were plated on sterile uncoated, rat tail collagen I-coated (Thermo Fisher Scientific) or A431 cell matrix-conditioned glass coverslips. For A431-conditioned cover slips, A431 cells were plated at confluence on glass cover-slips in 24 well plates, allowed to grow overnight, and removed by treating with PBS/EDTA at 37°C for 30 min followed by several washes with PBS/EDTA until coverslips were cleared. Cells cultured on coverslips were fixed with 10% formalin in HEPES-buffered saline with 4% sucrose and 1 mM MgCl_2_, rinsed twice with TBS, and blocked with 10% goat serum in PBS. For staining intracellular epitopes, cells were permeabilized with the addition of 0.1% NP-40 during the blocking step. Cells were stained for 1 h with primary antibodies in blocking buffer, briefly washed four times with TBS containing 0.1% Tween 20 (Sigma-Aldrich) (TBST), followed a 45 min treatment with the appropriate fluorescently labeled secondary antibodies in blocking buffer. For F-actin staining, Alexa Fluor 594 phalloidin (Thermo Fisher Scientific) was included with the secondary antibody in TBST containing 0.1% NP-40 instead of blocking buffer. For nuclear staining, 0.5 µg/ml DAPI (Sigma-Aldrich) was included in the secondary antibody step. After four additional washes with TBST, coverslips were mounted with Prolong Gold (Thermo Fisher Scientific) and analyzed by fluorescence microscopy. For immunostaining of tumor spheroids, spheroids created as described below were embedded in 0.8 mg/ml rat tail collagen I in a 35 mm glass-bottomed dish. After 48 h of invasion, spheroids were fixed for 2 h at room temperature with 10% formalin in PBS, rinsed extensively with TBS, and blocked overnight in PBS with 10% goat serum. Primary antibody was added in block and incubated overnight at 4°C. Spheroids were rinsed extensively with PBS, with the last rinse overnight at 4°C. Secondary antibody was added in block and incubated overnight at 4°C, followed by rinsing as for the primary antibody. Following the last rinse, spheroids were post-fixed for 2 h at room temperature with 10% formalin and then rinsed with TBS. Fluorescence images were acquired through the glass-bottomed dish using a Leica DMIRE2 inverted microscope with a 20× objective.

### Tumor spheroid assays

To create spheroids for 3D growth and invasion assays, 1×10^4^ cells per spheroid were plated in 96-well V-bottom plates that had been coated twice by air drying 25 µl of 20 mg/ml poly-HEMA (Sigma-Aldrich) in 95% ethanol in each well. Plates were spun briefly in a tabletop centrifuge to settle the cells, then cultured overnight in standard growth medium to allow spheroid formation. To create collagen gels for the invasion assay, 700 µl per well of 0.8 mg/ml rat tail collagen I in RPMI was allowed to polymerize for 1 h at 37°C in a 12-well plate. The plate was then placed on ice, and the spaces between the wells were filled with ice-cold PBS to maintain temperature during the transfer of spheroids. An additional 700 µl of ice-cold 0.8 mg/ml collagen I was overlaid on the cushion of pre-polymerized collagen I, and six spheroids per cell type were transferred to each collagen-containing well. To transfer, spheroids were recovered from the V-bottom wells by gently pipetting with a yellow tip to dislodge and capture the spheroids, and then spheroids were allowed to settle by gravity out of the tip and into the collagen, without ejecting any culture medium. After transfer, the collagen was polymerized for 1 h at 37°C, and wells were overlaid with 1 ml of serum-free medium. Spheroids were photographed using a Leica DMIRE2 inverted microscope with a 4× objective at the indicated time points. To compare spheroid integrity of parental, E-cadherin-expressing and E-cadherin-silenced cell lines, the spheroids formed as above were recovered by gentle pipetting and plated in 35 mm dishes for immediate photography.

### Time-lapse video microscopy

For random cell migration in 2D, a total of 1×10^5^ 4T1 parental cells were plated in standard growth medium on 35 mm culture dishes containing A431-conditioned substrate prepared as described above. The next day, the culture was maintained on a Leica DMIRE2 inverted microscope in a stage incubator (20/20 Technology, Wilmington, NC, USA) providing a humidified 5% CO_2_ and 37°C atmosphere. OpenLab software (Agilent, Santa Clara, CA, USA) running on an Apple iMac computer controlled illumination and image acquisition. Images were acquired at a rate of one frame every 3 min for 6 h, using a Hamamatsu ORCA-285 CCD camera and a 20× C Plan phase objective.

For tumor spheroid invasion in 3D, spheroids created as described above were embedded in 0.8 mg/ml rat tail collagen I in 35 mm glass-bottomed dishes, instead of in 12-well plates. Cell invasion was monitored using a Leica DMIRE2 inverted microscope with a 4× objective at a rate of one frame every 5 min for 40 h.

### Breast cancer growth and metastasis *in vivo*

All animal procedures in this study were approved by the University of Iowa Animal Care and Use Committee, Iowa City, IA, USA (approval number 5031328). Isoflurane was used for procedures requiring anesthesia, and euthanasia was performed by CO_2_ inhalation followed by cervical dislocation. For 4T1 cell experiments, 6- to 8-week-old female BALB/c mice (National Cancer Institute, Frederick, MD, USA) were implanted with 5000 cells in a volume of 50 µl in the fourth mammary fat pad. Bioluminescence imaging (BLI) was conducted in an IVIS100 imaging system (Perkin Elmer, Shelton, CT, USA) after intraperitoneal injection of luciferin (100 µl of 15 mg/ml solution per 10 g mouse body weight; GoldBio, St. Louis, MO, USA) as described previously. Whole-body tumor growth rates were measured as follows: a rectangular region of interest was placed around the dorsal and ventral images of each mouse and total photon flux (photons/second) was quantified using Living Image software v2.50 (Perkin Elmer). The dorsal and ventral values were summed and mean BLI values for each group were plotted weekly. Primary tumor growth was also measured by caliper, and tumor volumes were calculated using the formula (L×W^2^)/2, where L and W are the length and width of the tumor. For semi-quantitative analysis of spontaneous metastasis to the lung, *ex vivo* BLI was conducted on lungs harvested at assay endpoint (day 28). To ensure the best possible uniformity of measurement conditions, mice were sacrificed in groups of two or three, and lungs were immediately harvested and imaged *ex vivo*. All lungs were imaged within 20 to 30 min after euthanasia. 4T1 cells colonizing the lungs were recovered by mincing the lungs with a sterile razor blade and digesting with 200 U/ml collagenase II (Worthington Biochemical, Lakewood, NJ, USA) in complete medium for 15 min at 37°C. Explanted cells were grown out under G418 selection for analysis of E-cadherin expression by cell surface labeling and immunoprecipitation, as described above. Some lungs were harvested at the endpoint and fixed in 4% paraformaldehyde overnight at 4°C, rinsed and transferred to 30% ethanol, and stored at 4°C for later histological analysis.

*In vivo* studies of 4T07 cells were carried out in compliance with Purdue University's Institutional Animal Care and Use Committee (IACUC) guidelines. To create pulmonary tumors, luciferase-expressing 4T07 cells (5×10^5^) were injected into the lateral tail vein of 4- to 6-week-old female BALB/c mice that were that were obtained from Jackson Laboratories (Bar Harbor, ME, USA). The growth of pulmonary tumors was observed using an Ami HT bioluminescence imager (Spectral Instruments, Tucson, AZ, USA) following intraperitoneal injection of luciferin.

### Histological analysis

Formalin-fixed paraffin-embedded tissues were routinely stained with Hematoxylin and Eosin (H&E) (Sigma-Aldrich), and immunohistochemistry was performed for E-cadherin and vimentin. Antigen unmasking of paraffin sections was performed (citrate buffer, pH 6) in a decloaker (Biocare Medical, Pacheco, CA, USA). Endogenous peroxidase activity was quenched with 3% hydrogen peroxide and Background Buster (Innovex Biosciences, Richmond, CA, USA) was used to block non-specific staining. For E-cadherin staining, sections were incubated with rabbit anti-E-cadherin (clone 24E10, 3195, Cell Signaling Technology, Danvers, MA, USA) at 1:400 for 2 h and then incubated for 30 min with Rabbit Envision HRP System reagent (Agilent). For vimentin staining, sections were incubated with anti-vimentin primary antibody (ab94527, Abcam, Waltham, MA, USA) at 1:2000 for 60 min, followed by incubation with a biotinylated anti-rabbit antibody (1:500, 111-065-114, Jackson ImmunoResearch, West Grove, PA, USA) and Vectastain ABC reagent (Vector Laboratories, Newark, CA, USA). Slides were developed with DAKO DAB Plus (Agilent) for 5 min followed by DAB Enhancer (Agilent) for 3 min, before counterstaining with Hematoxylin. Slides were visualized using an Olympus BX5 microscope.

## Supplementary Material

10.1242/dmm.050771_sup1 Supplementary information
