# Supplementary material for: The highly metastatic 4T1 breast carcinoma model possesses features of a hybrid epithelial/mesenchymal phenotype
Source: Dis Model Mech. 2024 Sep 4;17(9):dmm050771. doi: 10.1242/dmm.050771 (PMC11391819; doi:10.1242/dmm.050771)
Supplement: Supplementary information [file dmm-17-050771-s1.pdf]

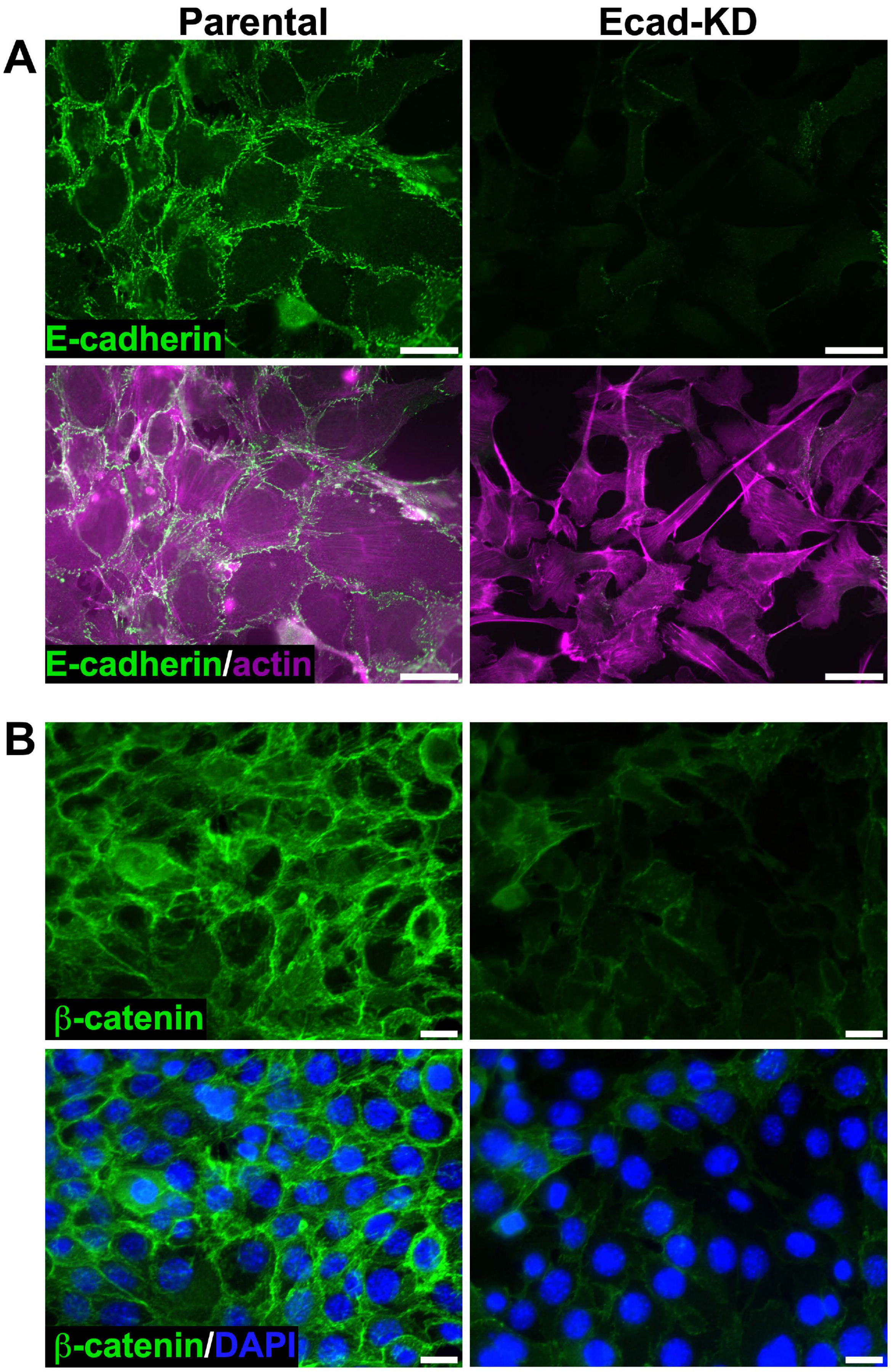

Fig. S1. **Loss of β-catenin in E-cadherin knockdown cells.** Parental and Ecad-KD cells were cultured on cover slips overnight. Cells were fixed, permeabilized and immunostained for (A) E-cadherin or (B) β-catenin antibodies, together with (A) AlexaFluor 594-conjugated phalloidin (to reveal F-actin) or (B) DAPI (to stain DNA). The level of β-catenin appeared much reduced in the E-cad KD cells compared to parental cells, and the remaining β-catenin did not obviously re-localize to the cell nucleus. Scale bars: 30 µm.

Disease Models & Mechanisms • Supplementary information

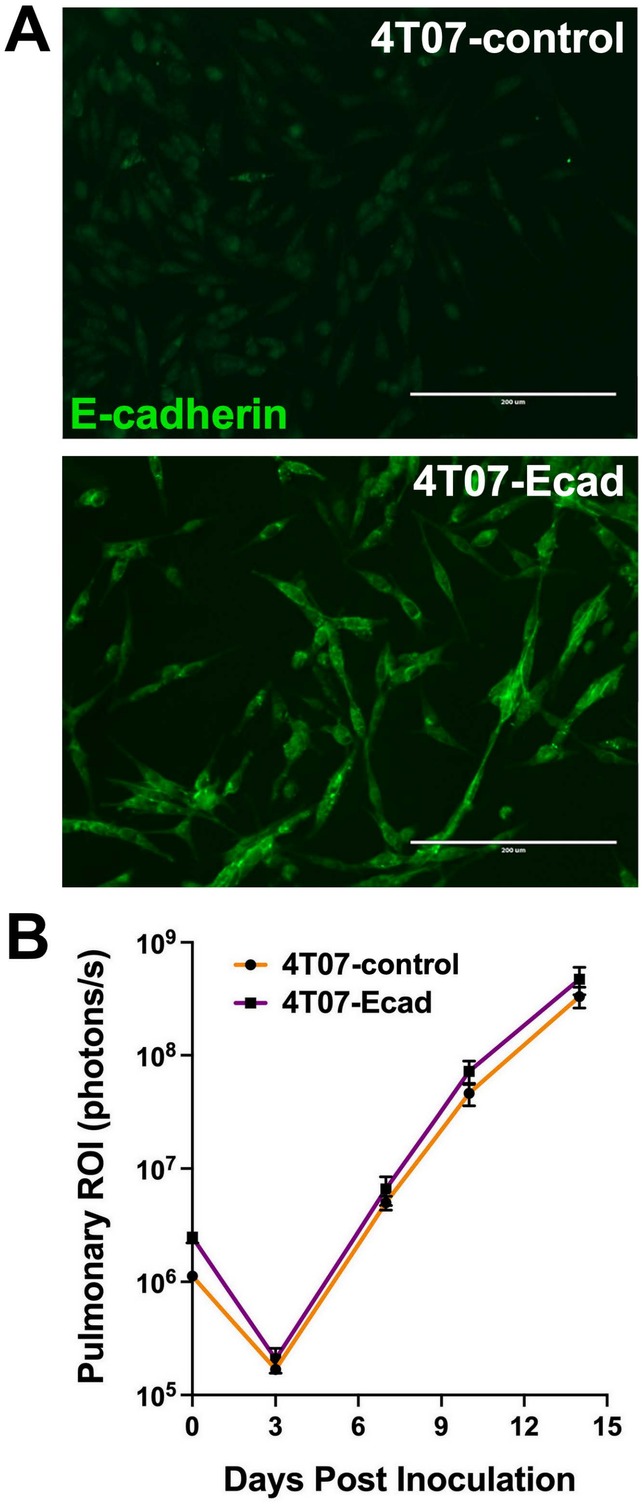

Figure S2

Fig. S2. E-cadherin overexpression does not alter 4T07 cell metastatic colonization. (A) 4T07 control cells or 4T07-E-cadherin overexpressing cells (4T07-Ecad) were immunostained for E-cadherin. Scale bar: 200 μm. (B) Luciferase-expressing 4T07 control or 4T07-Ecad cells were injected by tail vein into 5 mice/group (5 X 10^5 cells/mouse). Lung colonization was monitored over time via bioluminescence imaging within the pulmonary region of interest (ROI) of each mouse. No significant differences in lung colonization were observed (mixed-effects analysis with Tukey's multiple comparison test). Bars in B show the mean±s.e.m.

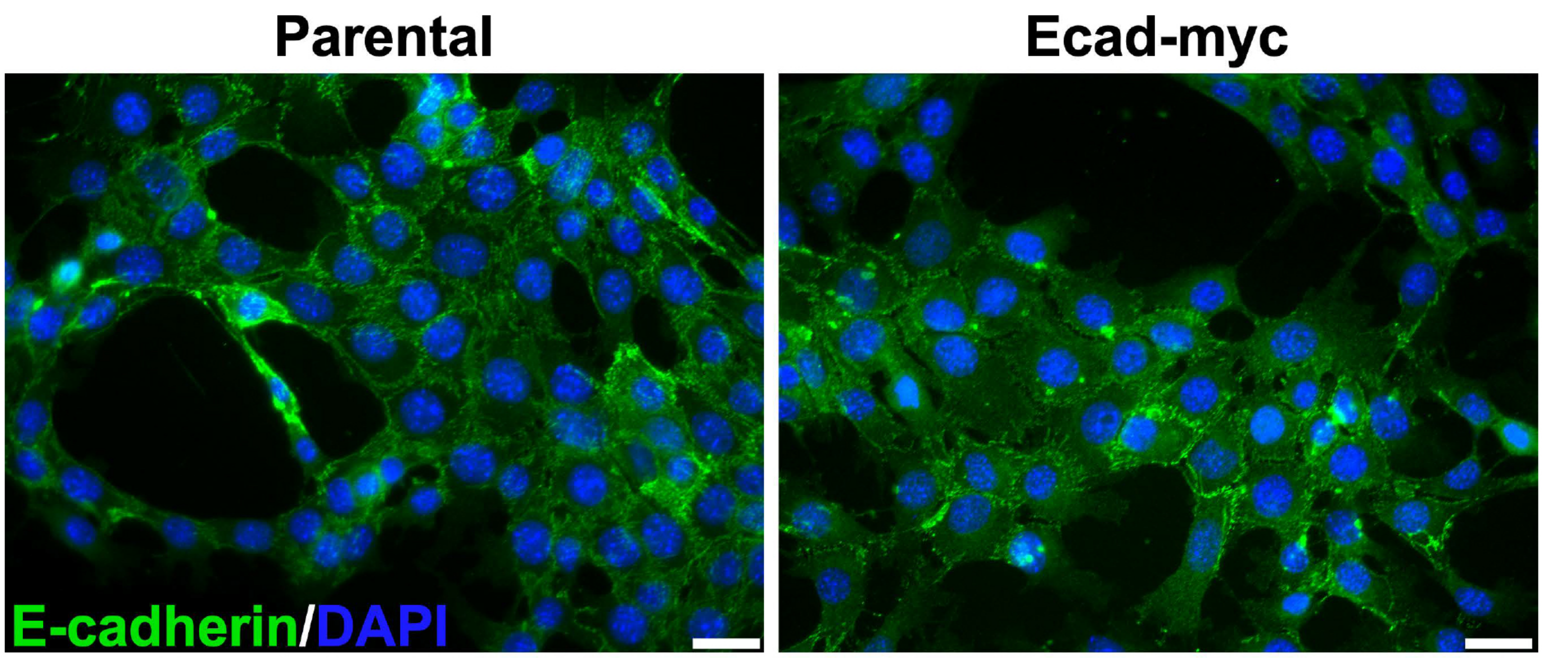

Fig. S3. E-cadherin-based cell-cell junctions are similarly perturbed in 4T1 parental and 4T1 Ecad-myc cells. 4T1 parental and 4T1 Ecad-myc cells were immunostained for E-cadherin and counterstained with DAPI. Cell-cell junctions were jagged and irregular in both cell types, as previously observed in 4T1 parental cells, and both cell types expressed a similar amount of total E-cadherin. Scale bars: 30 µm.

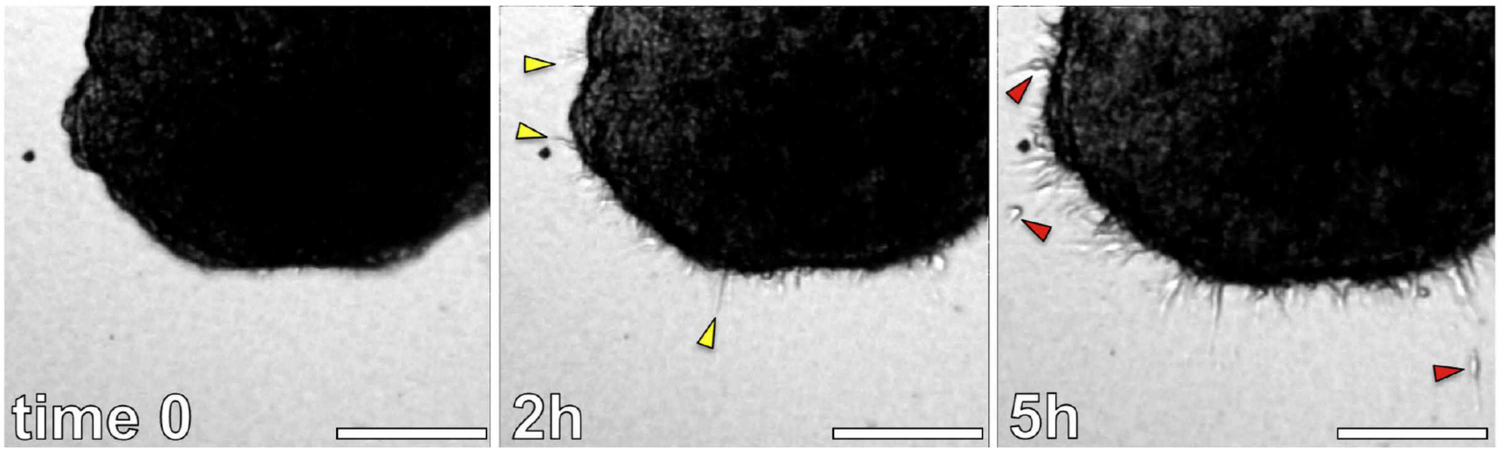

Fig. S4. 4T1 spheroids display early onset single cell invasion in 3D collagen. 4T1 spheroids were prepared as described in Materials & Methods. Micrographs of the edge of a spheroid are shown in which the image was taken immediately after embedding, and at two and five hours after embedding. The yellow arrowheads in point to multicellular structures oriented toward the matrix at the 2-hour time point. Red arrowheads point to single cells that have emerged from the spheroid by the 5-hour time point. This sequence illustrates how some individual cells can rapidly dissociate from the spheroid within hours after embedding in a collagen matrix. Scale bars: 200 µm.

Disease Models & Mechanisms • Supplementary information

Table S1. Primary antibodies used in this study

Target of antibody	Source	Antibody clone	Catalog No.	Type	Reactivity[1]	Application in this study[2]
E-cadherin	Sigma-Aldrich	DECMA-1	MABT26	Rat IgG	H M D	Flow, IF, IP (all 1:200)
E-cadherin	Thermo Fisher Scientific	Clone 36, BD	BDB61081	Mouse IgG	H M Rt D	IF (1:500), WB (1:2500)
E-cadherin	Cell Signaling Technology	24E10	3195	Rabbit IgG	H M	IHC (1:400)
N-cadherin	Cell Signaling Technology	D4R1H	13116	Rabbit IgG	H M	WB (1:1000)
β-catenin	Thermo Fisher Scientific	Clone 14, BD	BDB610154	Mouse IgG	H M Rt D	WB (1:500), IF (1:200)
p120-catenin	Thermo Fisher Scientific	Clone 98, BD	BD610134)	Mouse IgG	H M Rt D	WB (1:1000)
Vimentin	Abcam	EPR3776	ab92547	Rabbit IgG	H M Rt	IHC (1:2000)
Vimentin	Cell Signaling Technology	D21H3	5741	Rabbit IgG	H M	WB (1:1000)
Snail	Cell Signaling Technology	C15D3	3879	Rabbit IgG	H M Rt	WB (1:1000)
Zeb1	Cell Signaling Technology	E2G6Y	70512	Rabbit IgG	H M Rt	WB (1:1000)
tubulin	Developmental Studies Hybridoma Bank	12G10	12G10	Mouse IgG	H M Rt D	WB (1:500)
β-actin	Biolegend	Poly6221	622102	Rabbit IgG	H M Rt	IB (1:500)

[1]H, Human; M, Mouse; D, Dog; Rt, Rat
[2]Flow, flow cytometry; IF, immunofluorescence; IP, immunoprecipitation; IHC, immunohistochemistry; WB, western blotting

Disease Models & Mechanisms • Supplementary information

Table S2. Studies supporting a hybrid E/M phenotype in the 4T1 Breast Carcinoma Model

Epithelial/Mesenchymal Regulator or Marker[1]	Outcome of functional intervention in the 4T1/4T07 breast cancer models
Twist (drives mesenchymal phenotype)	Depletion in 4T1 cells *suppressed* spontaneous metastasis (Yang et al., 2004)
miR-9 (targets E-cadherin mRNA)	Downregulation in 4T1 cells *suppressed* spontaneous metastasis (Ma et al., 2010a)
miR-10b (upregulated by Twist)	Downregulation in 4T1 cells *suppressed* spontaneous metastasis (Ma et al., 2010b)
Zeppo1/ZNF703 (can trigger EMT)	Depletion in 4T1 cells *suppressed* spontaneous metastasis (Slorach et al., 2011)
miR-200 family (downregulates Zeb1, enforces epithelial phenotype)	Highly expressed in 4T1 cells. Over-expression in 4T07 cells *enhanced* spontaneous & experimental metastatic colonization (Korpal et al., 2011; Dykxhoorn et al., 2009)
ESRP-1/2 (epithelial splicing factors)	Depletion of ESRP-1 in 4T1 cells reduced CD44v expression and *suppressed* spontaneous metastasis (Yae et al., 2012)
E-cadherin	Depletion or forced expression in 4T1 cells had minimal impact on primary tumor growth or spontaneous metastasis (this study)<br>E-cadherin depletion modestly reduced metastatic outgrowth at early time points (Elisha et al., 2018)<br>An E-cadherin-depleted 4T1 subclone showed impaired primary tumor growth (Chu et al., 2013)<br>Preliminary data indicated that CRISPR deletion of E-cadherin in 4T1 cells had no effect on metastatic growth (Spencer et al., 2015)
FBXO11 (promotes degradation of Snail, which may be associated with a hybrid E/M phenotype)	Depletion of FBXO11 increased Snail expression in 4T1 cells and *promoted* 4T1 cell metastasis; over-expression of FBX011 had the converse effect (Zheng et al., 2014)
miR-155 (prevents EMT)	Forced expression of miR-155 in 4T1 *suppressed* spontaneous metastasis from fat pad but *promoted* experimental metastasis after tail vein injection (Xiang et al., 2011)
OVOL2 (may stabilize hybrid E/M state when expressed at endogenous level)	Forced over-expression increased epithelial phenotype of 4T1 and *suppressed* spontaneous lung metastasis (Wu et al., 2017)
GRHL2 (may stabilize hybrid E/M state when expressed at endogenous level)	Depletion of GRHL2 *suppressed* 4T1 cell lung metastasis (Wang et al., 2023)

[1]Mesenchymal markers are shaded **orange**, epithelial markers are shaded **blue**, and phenotypic stability factors are shaded **green**.

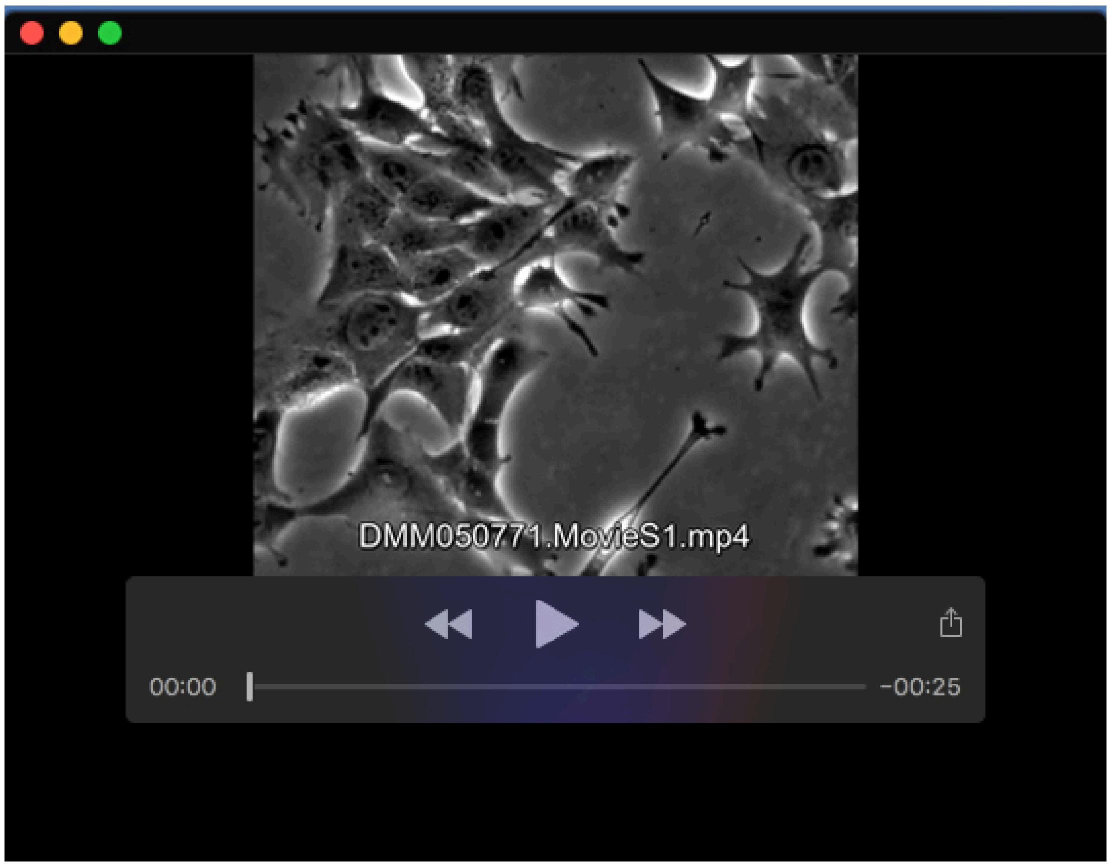

Movie 1. 4T1 cell junctions are highly dynamic. This video captures 4T1 cells making and breaking cell-cell contacts over a six-hour period, with one frame every three minutes. The play rate is eight frames/sec (1,440 X real time). Six still shots from this video correspond to Fig. 5 in the manuscript.

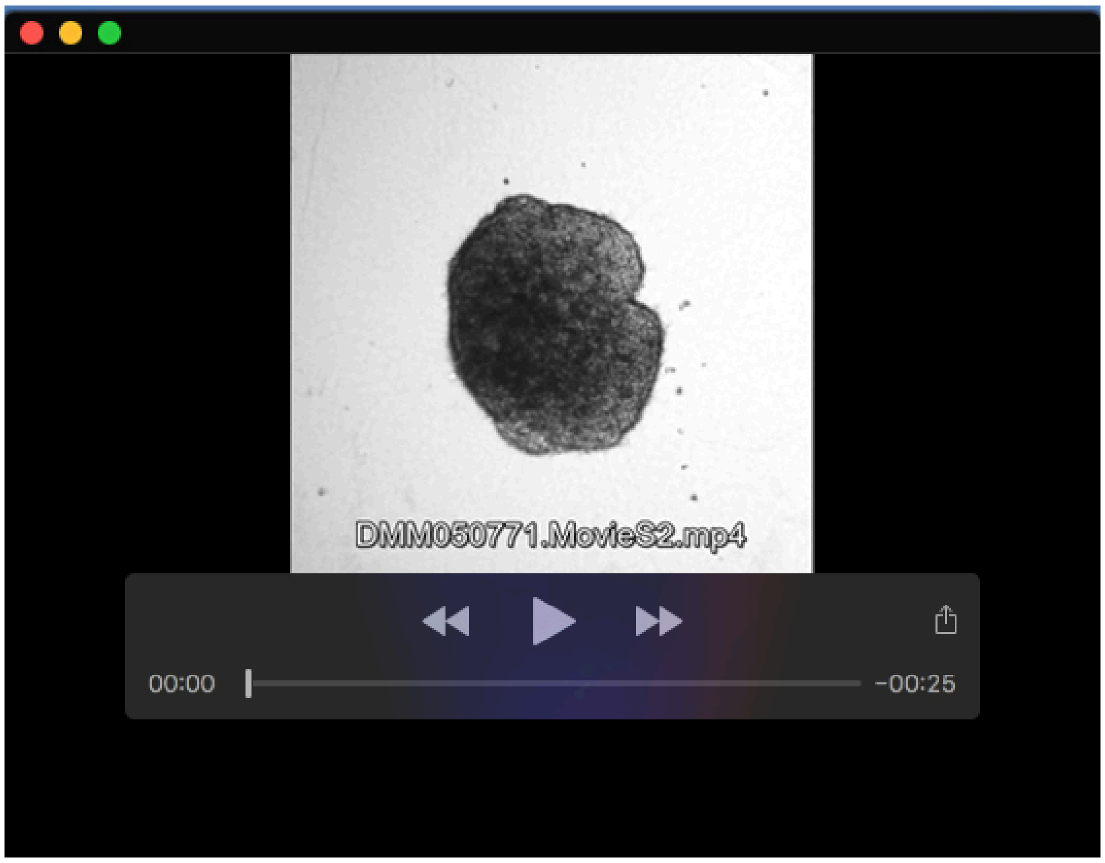

Movie 2. 4T1 cells within a spheroid invade in 3D culture conditions. This video shows 4T1 cell invasion into a 3D collagen matrix over a 40-h period, with one frame every five minutes. The play rate is eight frames/sec (2,400 X real time). A still shot from this video corresponds to Fig. S4 in the manuscript.

Disease Models & Mechanisms • Supplementary information

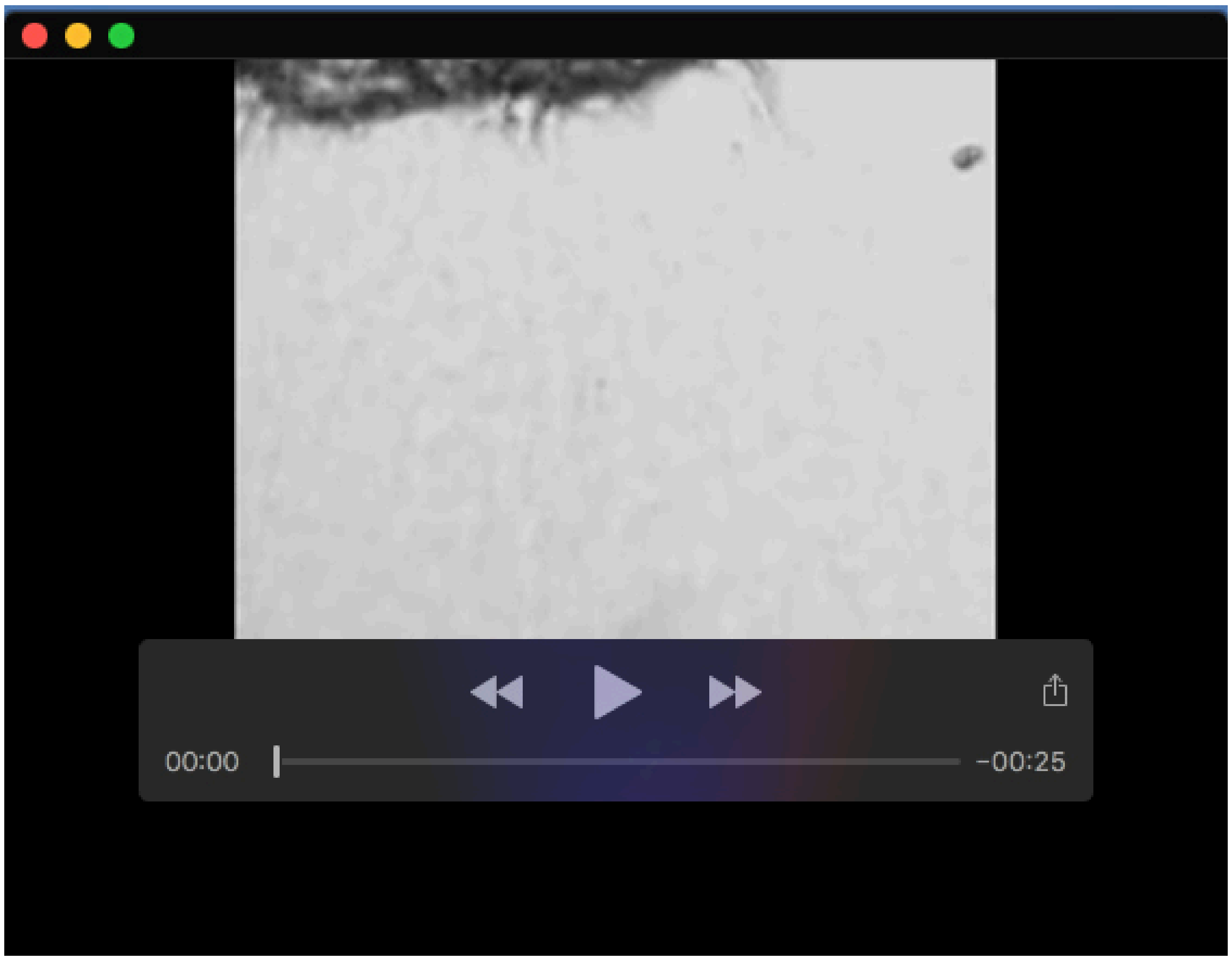

Movie 3. A magnified view of 4T1 cells within a spheroid invading in 3D culture conditions. This video is a zoomed and cropped view of Movie 2 centered around the bottom edge of the spheroid. The play rate is 16 frames/sec (4,800 X real time). Arrows in the final frame indicate cells that correspond to the immunostained image in Fig. 7 of the manuscript.
